# Low-cost UWB CPW microwave tattoo sensor for respiratory monitoring using near-field phase variation

**DOI:** 10.1038/s41598-026-58536-5

**Published:** 2026-06-30

**Authors:** Hadeer Ashraf, Anwer S. Abd El-Hameed, Islam Mansour, Gehan S. Shehata

**Affiliations:** 1 Electrical Engineering Department, Shoubra Faculty of Engineering, Benha University, Cairo, 11629 Egypt; 2 Microstrip Department, Electronics Research Institute (ERI), El Nozha, Cairo, 4473221 Egypt

**Keywords:** CPW slot sensor, Flexible UWB sensor, Gold leaf, Respiratory monitoring, SAR evaluation, Tattoo sensor, Ultra-wideband, Wearable biomedical devices, Engineering, Health care, Medical research

## Abstract

This paper presents a flexible, ultra-thin, and transparent tattoo-based CPW slot microwave sensor designed for respiratory monitoring with potential applications in epilepsy-related assessment. The sensor is fabricated using gold leaf on transparent PVC, enabling excellent skin conformity and long-term comfort. The proposed microwave sensor achieves an ultra-wide operating bandwidth of 2.4–17 GHz. Mechanical reliability is confirmed through bending and crumpling tests, where the sensor maintains stable impedance characteristics. In addition to respiratory monitoring, the proposed sensor shows potential for epilepsy-related monitoring applications, as epileptic seizures are often accompanied by abnormal respiratory patterns such as apnea, irregular breathing, or sudden changes in breathing rate. Continuous monitoring of respiration can therefore provide an indirect, non-invasive indicator of abnormal respiratory patterns associated with seizure activity. Experimental measurements on two healthy adult volunteers demonstrate clear respiratory-phase detection, with breathing rates of approximately 16 breaths per minute (BPM) for the first participant and 21 BPM for the second participant, which fall within the normal respiratory range for healthy adults under non-pathological conditions. Safety evaluation shows that the SAR values remain well below international exposure limits. The maximum simulated SAR values of 0.946 W/kg (1 g) and 0.258 W/kg (10 g) occur at 6 GHz and a transmitted power of 20 dBm, while significantly lower SAR levels are observed at lower frequencies and power levels. With its wide bandwidth, flexibility, skin transparency, and confirmed electromagnetic safety, the proposed sensor demonstrates strong potential for continuous healthcare monitoring, respiratory monitoring with potential relevance to epilepsy-related assessment, and future integration into unobtrusive wearable systems.

**Supplementary Information:**

The online version contains supplementary material available at 10.1038/s41598-026-58536-5.

## Introduction

Wireless communication technologies have become essential in modern life, enabling seamless connectivity across applications such as mobile communications, Internet of Things (IoT), smart healthcare, and real-time monitoring systems^1^. In particular, wireless sensing and communication techniques have opened new opportunities in biomedical applications by allowing continuous, remote, and non-invasive monitoring of physiological parameters. Advances in compact, low-power, and wearable wireless devices have further accelerated the development of healthcare monitoring systems that improve patient mobility, comfort, and quality of care^2^.

**Fig. 1:**
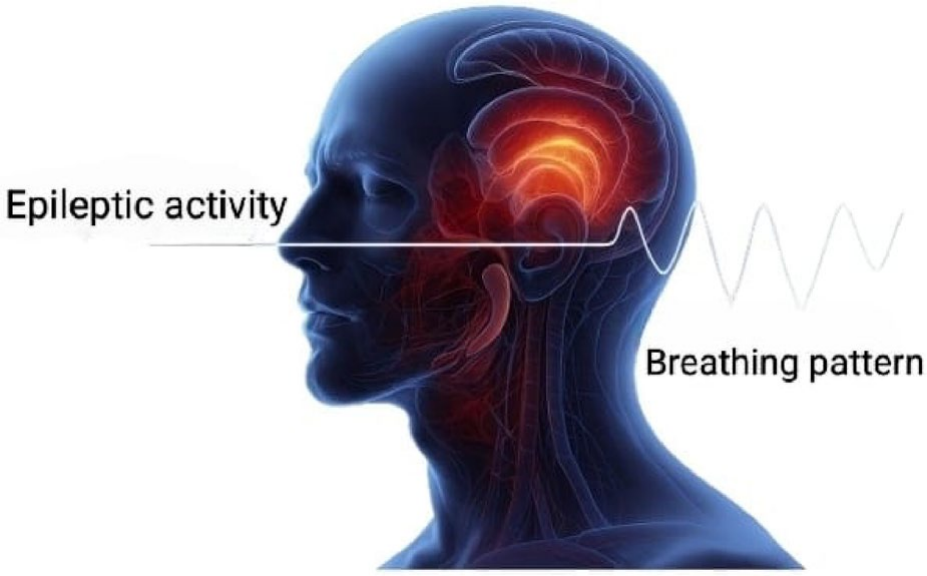
Epileptic seizure detection using breathing rate.

The increasing prevalence of chronic illnesses and the rising proportion of elderly individuals have intensified the global demand for remote healthcare solutions. Remote monitoring systems that track vital signs such as heart rate and respiration enable timely diagnosis, early intervention, and continuous patient assessment without requiring hospitalization^3^, Fig. 1. Wireless sensing technologies offer a particularly promising approach, allowing for real-time data collection via embedded or wearable devices^4^.

Epileptic seizures often occur without warning, posing risks of injury or medical emergencies. Research suggests that sudden changes in respiration may precede seizure onset, making respiratory rate a potential early warning indicator^5^. Non-invasive monitoring of such signs in daily life could significantly improve safety and care for epilepsy patients. Most existing seizure detection systems primarily rely on electroencephalography (EEG), which is a non-invasive technique for monitoring brain activity using electrodes placed on the scalp^6^. Although accurate in clinical environments, EEG systems often require multiple electrodes, conductive gel, and controlled settings, which make them impractical for continuous, real-life, or wearable applications. Additionally, some wearable seizure detection devices depend on motion or electrodermal activity, but these may not detect non-motor seizures and can suffer from false positives^7^. Therefore, there is a growing need for more comfortable, low-cost, and wearable solutions that can identify early physiological changes, such as abnormal respiratory patterns that may precede seizures, serving as early warning indicators.

Respiratory monitoring techniques typically include airflow sensors, chest bands, or accelerometers, which must remain in close contact with the body. While effective in clinical settings, these methods are often bulky or uncomfortable, limiting their practicality for continuous at-home monitoring. In contrast, a flexible sensor layer applied directly to the chest via transparent PVC offers a minimally obtrusive solution. These sensors can detect subtle chest movements or phase variations in transmitted radio signals, enabling accurate and comfortable respiratory tracking over extended periods^8^. Wearable electronics have transformed health monitoring by enabling continuous, real-world tracking of physiological parameters. They provide non-invasive and user-friendly platforms for long-term data collection^9^. Despite advances in wearable devices for heart rate monitoring, systems capable of accurate, continuous respiration tracking, especially in ambulatory environments, remain limited^10^. Furthermore, most commercial wearable devices, such as smartwatches and fitness bands, are designed primarily for consumer wellness rather than clinical accuracy. Their rigid form factor, limited sensing capabilities, high power consumption, and motion artifacts reduce their reliability for continuous medical use^11^. Additionally, current seizure detection systems often rely on EEG headsets or motion sensors, which are bulky, uncomfortable, or offer delayed response times^12^. A variety of sensor types, including flexible electrodes, textile-integrated sensors, stretchable photoplethysmography (PPG) modules, and radar-based systems, have been introduced to improve sensing accuracy and user comfort. For instance, ultra-thin PPG sensors and capacitive strain sensors have been applied to monitor heart and respiratory rates with improved signal fidelity during movement^13,14^. Additionally, wearable RF-based platforms have emerged as promising candidates for contactless monitoring by leveraging changes in electromagnetic signal reflection and transmission to detect motion due to breathing and cardiac activity^15,16^. Despite these advancements, current wearable sensors face several limitations. Many devices remain dependent on rigid substrates or bulky electronics, reducing comfort and wearability over long durations. Textile-based sensors may suffer from degradation due to environmental exposure, while PPG and accelerometer-based systems are sensitive to motion artifacts, impacting signal quality during physical activity^17^. Furthermore, existing solutions for respiratory monitoring and seizure detection often require complex, power-intensive circuits or are limited in their ability to predict seizure onset based on indirect physiological signs such as breathing irregularities^18^.

Ultra-Wideband (UWB) sensors are particularly well-suited for biomedical sensing due to their short-pulse, high-resolution signal capabilities and low power consumption. They are less prone to interference, provide better penetration through biological tissues, and offer accurate detection of small movements such as those caused by respiration or heartbeat^19^. Compared to narrowband (NB) sensors, UWB designs exhibit higher signal fidelity, broader bandwidth, and improved sensitivity, making them ideal for applications such as real-time respiration tracking and potential epilepsy-related monitoring^8^. Various UWB antenna configurations have been explored in recent literature, including monopole antenna, planar inverted-F antennas (PIFA), Vivaldi antennas, and dielectric resonator antennas (DRAs). While monopole antennas are simple and compact, they often suffer from limited radiation directionality and can be sensitive to detuning when placed on the human body^20^. Planar and wearable sensors integrated into textiles or flexible substrates have improved body conformity, yet frequently exhibit degraded impedance matching, limited gain, and high fabrication cost particularly when advanced conductive materials or multilayer fabrication are involved^21,22^. Vivaldi and DRA-based UWB sensing systems offer excellent bandwidth and directional radiation patterns but are typically bulky or rigid, making them unsuitable for skin-mounted applications^23^. Moreover, in many reported wearable designs, maintaining performance stability under bending, crumpling, or body movement remains a significant challenge. These limitations hinder their reliability in continuous biomedical monitoring scenarios where flexibility, low profile, and stable radiation behavior are essential.

To address the limitations of existing wearable respiratory monitors and their application in seizure detection, we introduce a novel, skin-conformal UWB sensor system designed for real-time respiratory monitoring, with the potential to function as a potential pre-seizure indication mechanism for epilepsy patients. The sensor is based on a coplanar waveguide (CPW) slot configuration, which offers key advantages including single-layer fabrication, a low profile, and ease of integration with planar circuits. This geometry ensures stable broadband performance while reducing sensitivity to proximity and deformation when worn on the body. By employing gold leaf as the conductive material and transparent PVC as the substrate, the design achieves excellent skin conformity, flexibility, and visual discretion, all at a fraction of the cost of conventional rigid or textile-based sensors. This low-cost fabrication method also simplifies prototyping and supports rapid iteration for biomedical applications. The proposed sensor operates over a wide impedance bandwidth from 2.4 to 17 GHz, maintaining a reflection coefficient better than − 10 dB across the band. In the proposed system, two identical sensors are placed on the chest, one acting as a transmitter and the other as a receiver and respiratory activity is captured by analyzing phase variations in the S21 parameter. This configuration enables the detection of respiratory variations, which may serve as a potential indicator of abnormal physiological conditions. Its application to epilepsy detection remains a subject for future investigation and requires clinical validation. Importantly, the sensor maintains a specific absorption rate (SAR) well within IEEE safety limits, with SAR values not exceeding 0.150 W/kg averaged over 1 g of tissue and 0.038 W/kg averaged over 10 g of tissue at 6 GHz and a transmitted power of 10 dBm, confirming its suitability for safe, continuous wear. It should be noted that the present study is limited to respiratory monitoring experiments conducted on a healthy subject. No seizure-related data were collected, and the discussion of epilepsy is intended to highlight a potential future application of the proposed system.

The rest of this paper is organized as follows. Section 2 presents the sensor design, including the configuration, structure, design stages, specifications, simulation setup, parametric analysis. Section 3 describes the fabrication procedure of the proposed sensor. Section 4 includes both simulated and measured results. Section 5 investigates the effects of mechanical deformation specifically bending and crumpling on sensor performance. Section 6 evaluates the safety of the design through SAR analysis. Section 7 introduces the proposed respiratory monitoring system based on phase variation analysis, detailing the operating principle, performance in different body positions, and its potential applicability to epilepsy-related monitoring. Section 8 provides a comprehensive discussion and comparison, including a summary of findings, a comparative analysis with recent literature, and the strengths and limitations of the proposed design. Finally, Sect. 9 concludes the paper and highlights future research directions.

## Sensor design

The proposed sensor is developed for integration into wearable biomedical systems for continuous respiratory monitoring and seizure prediction. Key requirements for this application include UWB operation, a compact and flexible form factor, and stable electromagnetic performance when mounted on the human body. UWB performance is essential for achieving high resolution in detecting minor physiological changes^24^, while miniaturization and mechanical flexibility ensure comfort and usability in long-term, on-body conditions^22^.

### Sensor configuration and structure

The proposed sensor is a CPW-fed microstrip slot sensor designed with a Defected Ground Structure (DGS) to enhance impedance bandwidth and radiation characteristics^8^. The radiating element is the slot itself, which is etched into the ground plane in the form of a symmetric heart-shaped pattern. The initial dimensions of the proposed sensor were estimated based on the resonant frequency equation of a semi-circular slot radiator, given by Eq. (1). The lowest resonant frequency is related to the effective current path length surrounding the slot and can be expressed as:

1 $$\:{f}_{r}=\frac{c}{2{L}_{eff}\sqrt{{\epsilon\:}_{eff}}},{L}_{eff}\approx\:\pi\:r+2r$$

where $\:c$is the speed of light in free space, $\:{L}_{eff}$is the effective current path length, $\:r$is the radius of the semi-circular slot, and $\:{\epsilon\:}_{eff}$is the effective dielectric constant of the substrate. Based on Eq. (1), the initial radius was selected such that the sensor resonance starts near 2.4 GHz. After obtaining this initial geometry, several slot modifications and additional current paths were introduced to increase the effective electrical length, allowing the overall sensor dimensions to be reduced while maintaining the desired lower operating frequency. Finally, CST Microwave Studio was used to optimize the structure for impedance matching and ultra-wideband operation.

This continuous pattern introduces multiple current paths and resonant modes, enabling UWB behavior and stable radiation performance when integrated into wearable biomedical systems^25^. The proposed sensor is designed to operate over the ultra-wideband (UWB) range due to the advantages of UWB in wearable biomedical sensing applications, including higher resolution and improved sensitivity to small physiological movements^8^. In addition, the multiple resonances generated by the proposed structure overlap to achieve wide impedance bandwidth and improved matching across the operating band, ensuring stable and reliable sensing performance under on-body conditions. At the center of the CPW feedline, a heart-shaped tuning stub is embedded to finely adjust impedance matching and enhance the sensor’s resonant behavior. The combination of the symmetric heart-shaped slot, the central tuning stub, and the DGS-modified ground plane not only broadens the operational bandwidth but also ensures good radiation efficiency under body-mounted conditions^26^. The selection of the heart-shaped geometry is motivated by its smooth symmetric curvature, which promotes a more uniform current distribution and introduces multiple resonant paths, thereby enhancing impedance bandwidth and radiation stability compared to conventional slot geometries such as circular or rectangular shapes. This integrated configuration offers a compact, low-profile, and flexible structure well-suited for UWB biomedical and wearable communication applications, where both high performance and mechanical adaptability are critical^27^.

**Fig. 2:**
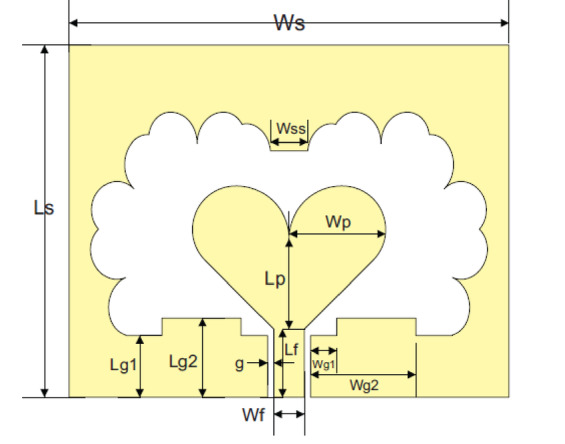
Configuration of the UWB sensor.

The proposed sensor achieves UWB performance from 2.4 to 17 GHz with |S₁₁| ≤ − 10 dB, fabricated on a flexible transparent PVC substrate of 0.3 mm thickness, ε = 2.7, and tan δ = 0.007, offering a compact footprint of 40 × 50 mm² and a CPW feedline of 7.75 mm length, 3.5 mm width, and 0.7 mm gap, with additional slot and ground shaping parameters presented in Fig. 2. and Table 1 to support stable broadband operation on the human body.

The motivation behind the proposed design stems from the requirements of wearable ultra-wideband biomedical sensing systems, where flexibility, compactness, and stable performance under human body loading are essential^9^. The CPW-fed microstrip slot configuration with a defected ground structure (DGS) is selected due to its simple fabrication, wide impedance bandwidth, and compatibility with flexible substrates^19^. The symmetric heart-shaped geometry is intentionally adopted to introduce multiple current paths and resonant modes, which enhances bandwidth and improves radiation stability under bending and on-body conditions^25^. In addition, the central tuning stub enables precise impedance matching across the ultra-wideband range^26^. Unlike conventional UWB antenna geometries reported in the literature, the proposed design is implemented on a low-cost transparent PVC substrate in a tattoo-like form factor and is specifically optimized for integration into a dual-sensor S21 phase-based respiration monitoring system, which represents the key novelty of this work^15^.

**Table 1:** The optimized dimensions of the proposed UWB sensor (all dimensions in mm).

$\:{L}_{s}$	$\:{\:\:L}_{p}$	$\:{L}_{g1}$	$\:{L}_{g2}$	$\:{L}_{f}$	g
40	10.25	7	9	7.75	0.7
$\:{W}_{s}$	$\:{W}_{p}$	$\:{W}_{g1}$	$\:{W}_{g2}$	$\:{W}_{f}$	$\:{W}_{ss}$
50	10.97	3	12	3.5	4.25

The electromagnetic performance of the proposed sensor was evaluated using full-wave simulations conducted in CST Studio Suite^28^. The software was used to analyze the sensor behavior over the ultra-wideband frequency range of 2.4–17 GHz, enabling accurate evaluation of key performance parameters such as the reflection coefficient (|S₁₁|), input impedance stability, and operational bandwidth. Throughout the iterative design process, simulated results were monitored to guide geometric optimization and ensure stable broadband performance suitable for wearable biomedical applications^29^.

### Design stages of sensor

**Fig. 3:**
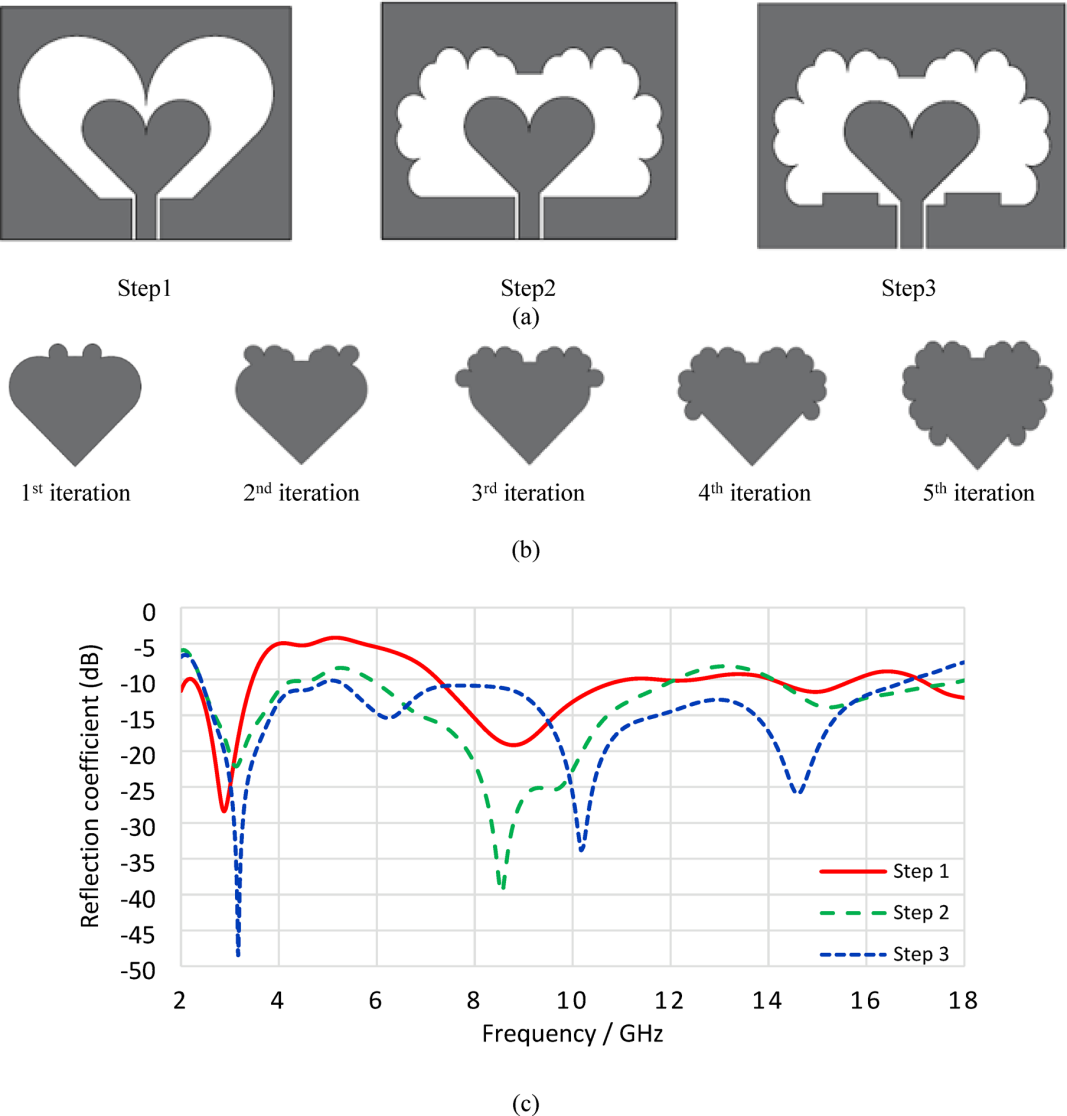
(**a**) Design steps, (**b**) Hart shape geometry in its different iteration stages, and (**c**) Simulated |S11| of design steps.

The sensor design evolved through three steps to enhance impedance matching and bandwidth, as illustrated in Fig. 3(a). In Step 1, a dual-band response was achieved using a heart-shaped slot, covering 2.25–3.45 GHz and 7.24–10.95 GHz with moderate matching. In Step 2, the slot was modified into a heart-shaped pattern in which repeated iterations are carried out as shown in Fig. 3(b), which significantly improved the reflection coefficient at 8.5 GHz from − 20 dB to − 40 dB and expanded the bandwidths to 2.41–4.68 GHz and 5.9–12.13 GHz. Although the geometry evolution visually resembles fractal-like patterns due to repeated edge modifications, it does not follow strict self-similar scaling. Instead, these iterations are employed to introduce additional current paths and multiple resonant modes, leading to bandwidth enhancement and improved impedance matching. In Step 3, further modifications were applied to the ground structure, resulting in three distinct resonant frequencies at 3.17 GHz, 10.17 GHz, and 14.6 GHz. This final step achieved improved impedance matching and a wide operational bandwidth extending from 2.4 to 17 GHz, as shown in the reflection coefficient responses in Fig. 3(c).

### Equivalent circuit analysis

#### Equivalent circuit evolution

**Fig. 4:**
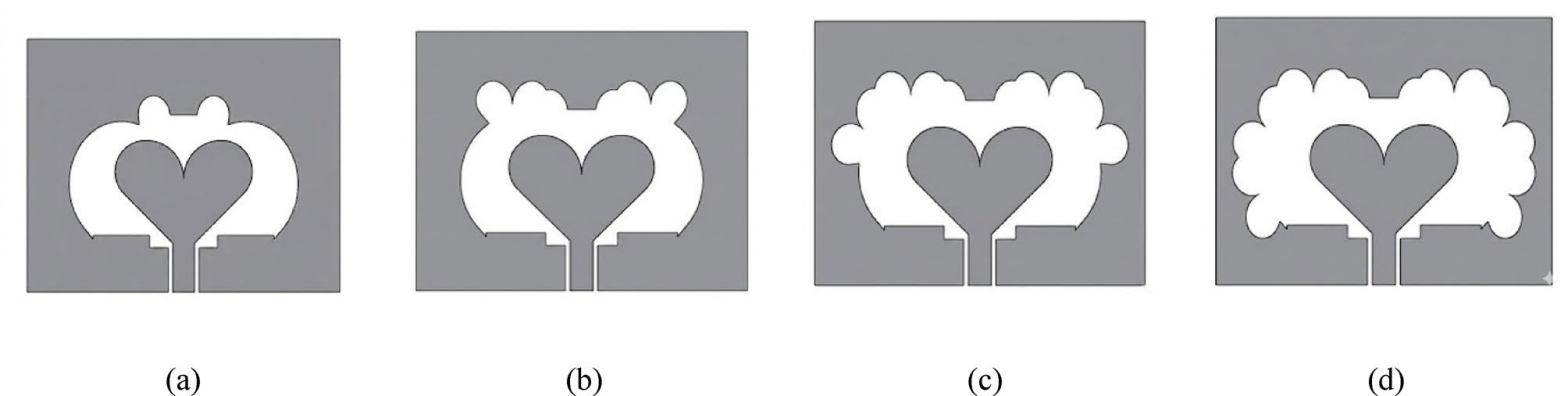
Evolution of the proposed antenna geometry: (**a**) Iteration 1, (**b**) Iteration 2, (**c**) Iteration 3, and (**d**) Iteration 4.

**Fig. 5:**
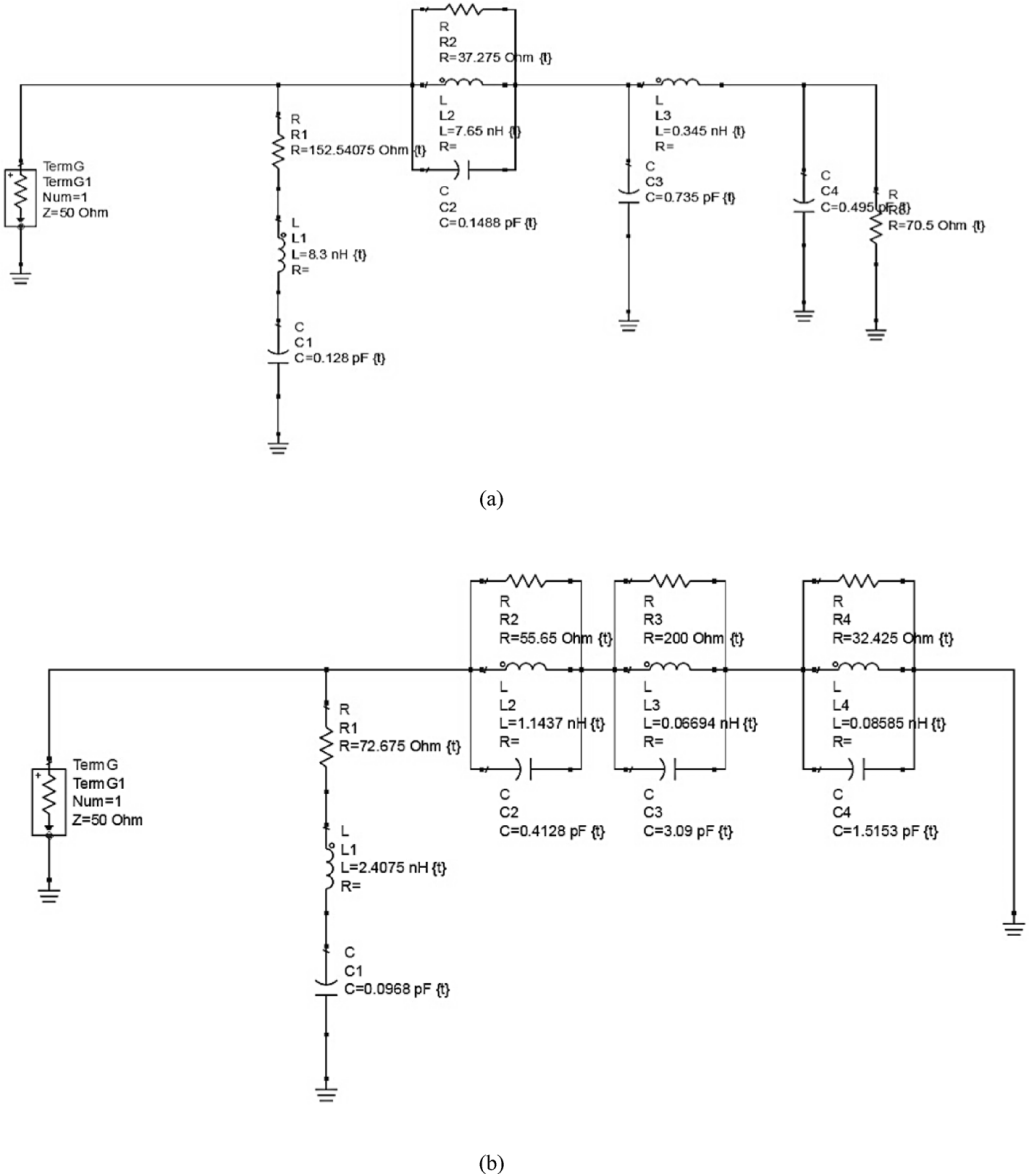

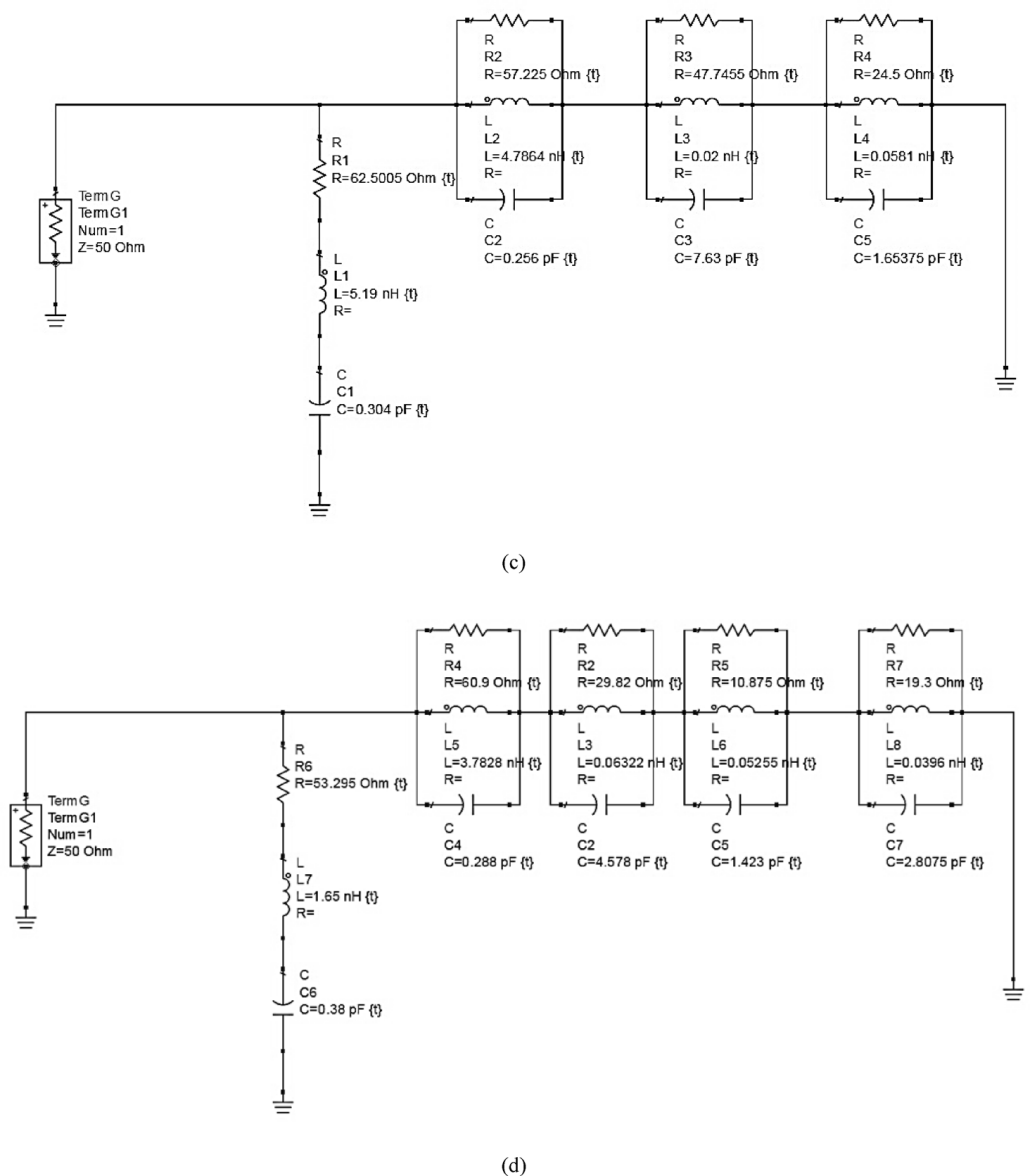
Equivalent circuit models of the antenna design iterations: (**a**) Iteration 1, (**b**) Iteration 2, (**c**) Iteration 3, and (**d**) Iteration 4.

**Fig. 6:**
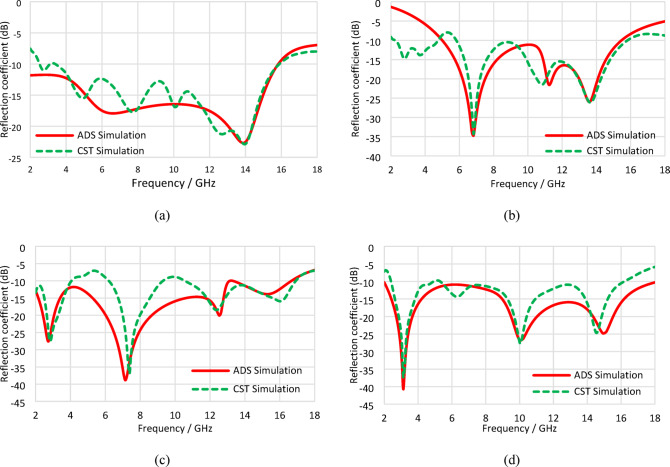
ADS and CST reflection coefficient comparison for the antenna design iterations: (**a**) Iteration 1, (**b**) Iteration 2, (**c**) Iteration 3, and (**d**) Iteration 4.

To establish the relationship between the geometric evolution of the proposed antenna and its resonant behavior, equivalent circuit models were developed for the four design iterations shown in Fig. 4. The corresponding equivalent circuits developed using ADS and ADS–CST reflection coefficient comparisons are presented in Figs. 5 and 6, respectively.

In the first Iteration, the basic heart-shaped radiator generates the fundamental resonant behavior of the antenna. The equivalent circuit consists of a single dominant parallel RLC resonator together with the feed-related reactive elements. As shown in Fig. 6(a), this configuration provides the initial wideband response; however, the impedance matching is not optimal over the entire operating band.

In the second Iteration, the heart-shaped geometry is further expanded by introducing additional conductive sections, which create new current paths and modify the coupling effects within the radiator. These modifications generate two additional resonant modes, which are represented in the equivalent circuit by the addition of two parallel RLC resonator branches. As a result, the impedance matching is significantly improved and the operating bandwidth is extended, as confirmed by the ADS and CST responses in Fig. 6(b).

In the third Iteration, the geometry is further modified without introducing new resonant paths. Therefore, the equivalent circuit topology remains unchanged, while only the values of the reactive elements are adjusted to reflect the altered current distribution and coupling conditions. These parameter variations shift the resonance locations and improve the matching characteristics, leading to a better agreement with the desired wideband response.

In the fourth Iteration, an additional geometric feature is incorporated into the radiator, producing another resonant mode within the operating band. This behavior is modeled by introducing an additional parallel RLC resonator branch in the equivalent circuit. The new resonance contributes to the formation of a third center frequency and further enhances the impedance bandwidth. The close agreement between the ADS and CST results demonstrates the capability of the equivalent circuit model to accurately represent the electromagnetic behavior of the antenna throughout the design evolution^30^.

**Fig. 7:**
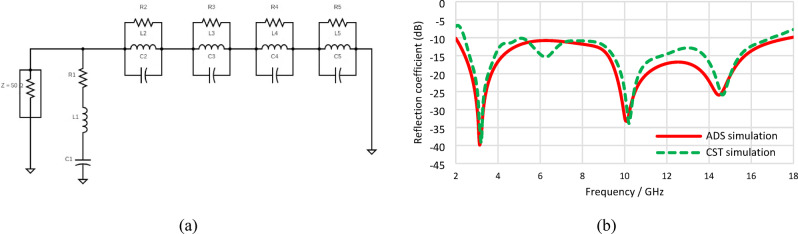
(**a**) Proposed equivalent lumped-element circuit model of the designed UWB sensor implemented in ADS. (**b**) Comparison of the simulated reflection coefficient s11obtained from the ADS equivalent circuit model and the CST full-wave simulation.

#### Final equivalent circuit and validation

Following the circuit evolution presented in Sect. 2.3.1, the final optimized equivalent lumped-element model of the proposed sensor is shown in Fig. 7(a). The model captures the combined resonant behavior of the optimized antenna and provides a circuit-based interpretation of its ultra-wideband operation. The model consists of a series RLC branch $\:\left({R}_{1},{L}_{1},{C}_{1}\right)$, representing the feedline effect and coupling between the CPW feed and the radiating slot structure. In addition, four parallel RLC resonator branches $\:\left({R}_{2},{L}_{2},{C}_{2}\right)$–$\:\left({R}_{5},{L}_{5},{C}_{5}\right)$ are connected in cascade to model the multiple resonant modes introduced by the heart-shaped slot iterations and the defected ground structure^30^. The resistive elements represent radiation and conduction losses, the inductive elements correspond to the current paths along the slot and ground edges, and the capacitive elements represent the coupling and fringing field effects between adjacent metallic regions. The values of the lumped components were extracted and optimized in ADS to obtain a reflection coefficient response close to that of the full-wave CST simulation. The optimized values of all equivalent circuit components are listed in Table 2. Figure 7(b) shows the comparison between the reflection coefficient obtained from ADS and CST. Good agreement is observed between the two responses, particularly at the main resonant frequencies, validating the proposed equivalent circuit model for representing the sensor’s ultra-wideband and multi-resonant behavior.

**Fig. 8:**
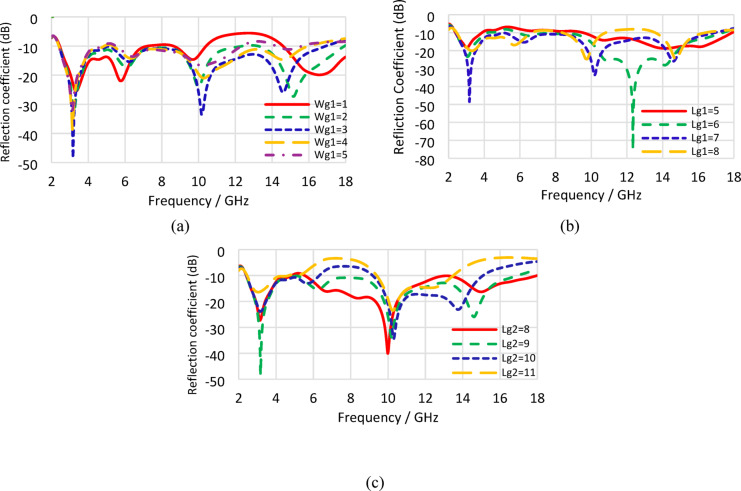
|S11| versus frequency for different parametric sweeps. (**a**) Effect of W_g1. (**b**)Effect of L_g1.(**c**) Effect of L_g2.

**Table 2:** Optimized lumped-element values of the proposed equivalent circuit model.

Parameter	Value	Parameter	Value
$\:{R}_{1}$	53.295 Ω	$\:{L}_{4}$	0.06 nH
$\:{R}_{2}$	60.9 Ω	$\:{L}_{5}$	0.04145 nH
$\:{R}_{3}$	29.82 Ω	$\:{C}_{1}$	0.38 pF
$\:{R}_{4}$	11 Ω	$\:{C}_{2}$	0.288 pF
$\:{R}_{5}$	19.3 Ω	$\:{C}_{3}$	4.5235 pF
$\:{L}_{1}$	1.65 nH	$\:{C}_{4}$	1.182 pF
$\:{L}_{2}$	3.9785 nH	$\:{C}_{5}$	2.7793 pF
$\:{L}_{3}$	0.06322 nH		

### Parametric analysis

The influence of key design parameters on the sensor’s impedance matching and resonant behavior is illustrated in Fig. 8(a–c). As shown in Fig. 8(a), increasing the horizontal length of the region etched from the ground plane ($\:{W}_{g1}$) improves impedance matching up to 3 mm; beyond this point, the matching deteriorates, particularly around 12 GHz. This behavior can be attributed to the role of the defected ground structure in modifying the surface current distribution. Increasing $\:{\boldsymbol{W}}_{\boldsymbol{g}1}$ enhances the capacitive coupling between the radiator and the ground plane, leading to improved impedance matching. However, excessive increase disturbs the current balance and degrades the matching at higher frequencies. Figure 8(b) demonstrates that as the vertical length of the etched ground ($\:{\mathrm{L}}_{\mathrm{g}1}$) increases, the impedance matching at the first resonance improves until it reaches 7 mm. This is due to the extension of the surface current path along the defected ground, which increases the effective electrical length and enhances energy coupling. However, further increase leads to reduced matching and the emergence of a third resonance near 7 mm. This additional resonance can be attributed to the excitation of higher-order modes resulting from excessive elongation of the ground slot. Similarly, Fig. 8(c) shows that increasing the vertical length of ground $\:{L}_{g2}$ enhances matching at the first resonance up to 9 mm, after which the performance declines and a third resonance appears. This behavior is associated with further modification of current paths and the introduction of additional reactive effects, resulting in multi-resonant characteristics. It is worth noting that the defected ground structure plays a critical role in controlling the sensor performance, as it modifies the current distribution, coupling mechanism, and effective electrical length, thereby enabling wideband operation.**Figure.8.** |S11| versus frequency for different parametric sweeps. (a) Effect of $\:{W}_{g1}$. (b)Effect of $\:{L}_{g1}$.(c) Effect of $\:{L}_{g2}$.

### Sensor fabrication

**Fig. 9:**
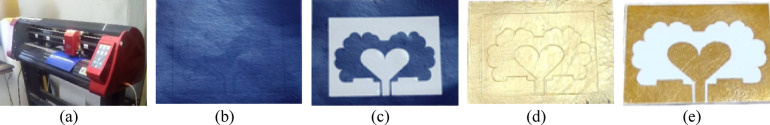
(**a**) Cutting design with cutter plotter. (**b**) PVC after cutting. (**c**) Creating stencils of the sensor. (**d**) Applying gold leaf as conducting material. and (**e**) After removing negative areas of the sensor.

This section details the low-cost and reproducible fabrication process of the proposed wearable UWB CPW sensor using transparent adhesive PVC and gold leaf^31^. Unlike conventional fabrication techniques such as inkjet printing, screen printing, or photolithography, which often require specialized equipment, controlled environments, or post-processing steps, the proposed method relies entirely on commercially available materials and consumer-grade tools. This eliminates the need for cleanroom facilities, high-temperature curing, or expensive conductive inks^32^. The process leverages readily available materials and minimal equipment, enabling fabrication without access to specialized facilities. The steps are summarized in the following subsections and illustrated in Fig. 9. The sensor is fabricated on an A4-sized transparent adhesive vinyl sheet (PVC), chosen for its flexibility, skin conformity, and ease of availability. In contrast to traditional rigid or opaque substrates, the use of transparent PVC enables a visually unobtrusive, tattoo-like sensor suitable for wearable biomedical applications. A layer of blue cut vinyl is adhered to the PVC surface to serve as a stencil for the sensor pattern. This dual-layer setup allows for precise pattern transfer and reliable support for gold leaf application. The sensor layout is designed using standard 2D design software and cut into the blue vinyl using a Sky Cut V24 cutter plotter. This approach allows rapid and repeatable fabrication with high geometrical accuracy using widely accessible digital cutting tools, making the method scalable and easily reproducible. This electronic cutter ensures sharp edges and accurate geometry of the CPW slot structure. Precision in this stage directly influences the quality of the resulting sensor. After cutting, the positive (unwanted) parts of the stencil are manually removed using tweezers or a weeding tool. This exposes the adhesive PVC areas corresponding to the sensor trace layout. These regions will be used for applying the conductive material. A thin layer of Pebeo Gedeo gilding paste glue is applied to the exposed adhesive surface using a brush. The glue is left for a few minutes until it becomes tacky. Unlike conductive ink-based methods, this approach avoids issues related to ink viscosity, nozzle clogging, and curing conditions, simplifying the fabrication workflow. Gold leaf sheets are gently applied onto the tacky glue. Each gold leaf sheet measures approximately 14 × 14 cm, while the sensor layout occupies a compact area of 5 × 4 cm, allowing multiple sensors to be fabricated from a single sheet. This significantly reduces material cost per device compared to conventional metallic deposition or printing techniques. The gold leaf adheres to the glued areas, forming conductive traces. Excess gold is brushed off with a soft tool. Gold leaf has recently gained attention in low-cost wearable and tattoo-based electronics due to its conductivity, biocompatibility, and aesthetic appeal. Once the glue is completely dry, the remaining negative parts of the vinyl stencil are removed. This leaves behind the final patterned gold leaf traces directly on the transparent PVC, forming the complete sensor. The resulting structure combines flexibility, transparency, ultra-low cost, and ease of fabrication, which are rarely achieved simultaneously in existing wearable sensor fabrication techniques. The final sensor is flexible, transparent, and visually discreet. It resembles a skin-like tattoo structure and conforms easily to body contours, making the design accessible and scalable for wearable biomedical applications^33^.

## Simulation and measurement results

**Fig. 10:**
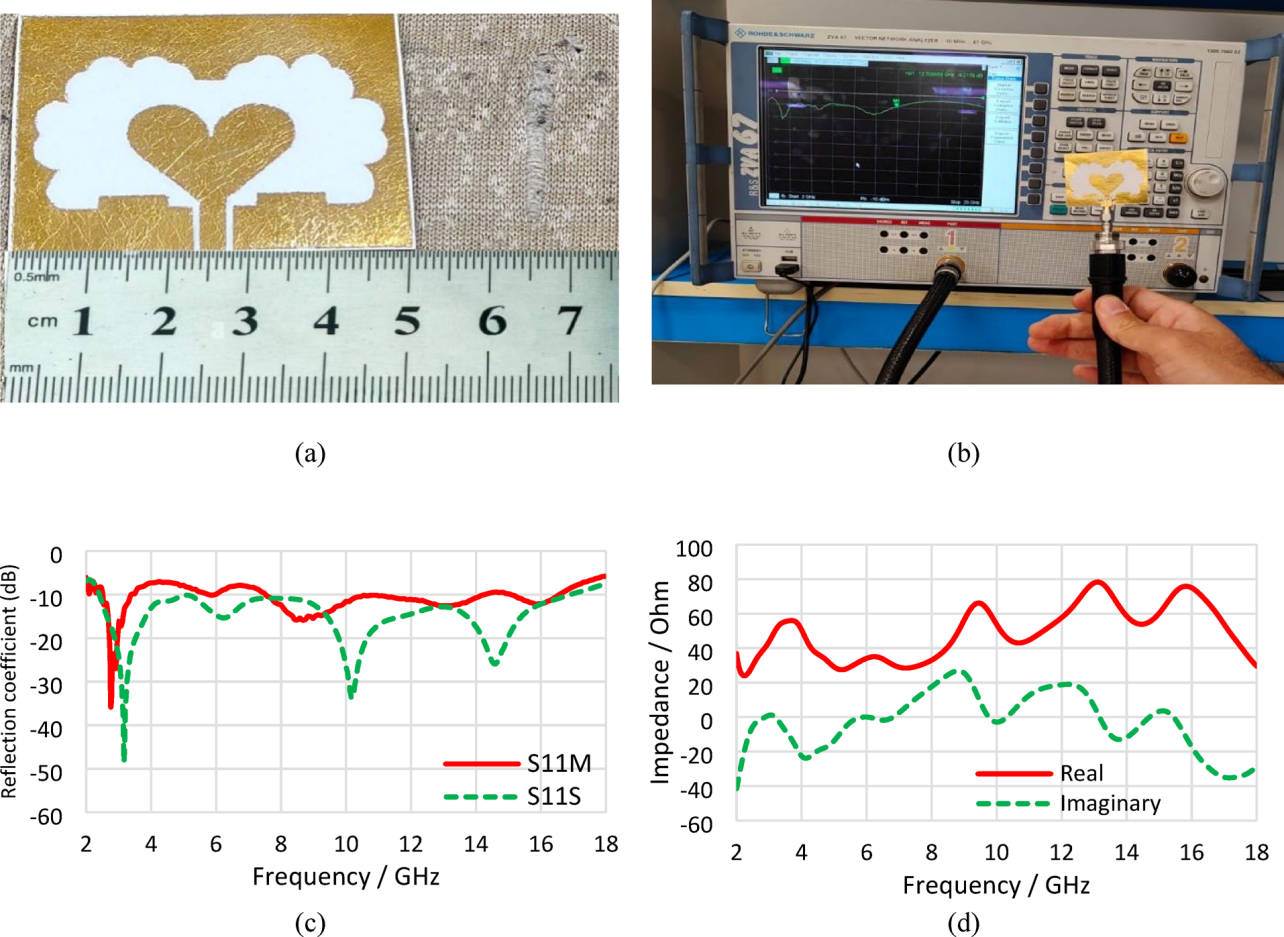
Measurement result. (**a**) Photo of the fabricated sensor. (**b**) Measurement setup. (**c**) Simulated and measured reflection coefficient. (**d**) Impedance (real and imaginary). (**e**) Simulated realized gain of the proposed sensor versus frequency. (**f**) Simulated total efficiency of the proposed sensor versus frequency.

The sensor’s impedance matching performance was assessed by comparing the simulated S11 and measured S11 reflection coefficient curves, as shown in Fig. 10(c). The measured S11 remained below the standard − 10 dB threshold across a very wide frequency range, spanning from approximately 2.2 up to 7 GHz, consistent with UWB flexible CPW-based sensor behavior. The measured S11 results show good agreement with the simulated data, with a noticeable shift toward lower frequencies. This shift is mainly due to an increase in the effective permittivity caused by the flexible PVC substrate, adhesive layer, and small air gaps, which collectively increase the effective electrical length of the sensor. In addition, fabrication tolerances, including dimensional inaccuracies during fabrication and soldering imperfections at the CPW feed, also contribute to the observed discrepancies. Such variations are commonly reported in flexible CPW-based UWB prototypes^33^.

Despite these practical factors, the measured S11 validated the ultra-wideband operation necessary for accurate respiratory sensing, aligned with UWB biomedical sensing principles^15^. The simulated input impedance components, $\:{Z}_{in}$=$\:{R}_{in}$+j$\:{X}_{\mathrm{i}\mathrm{n}}$ are detailed in Fig. 10(d). The real part fluctuated around the target 50 Ω characteristic impedance throughout the operational band. The imaginary part exhibited variations across the spectrum, confirming that the sensor wideband nature relies on balancing distributed capacitance and inductance inherent in CPW slot geometries to maintain a reflection coefficient ≤ − 10 dB, as discussed in CPW-fed UWB sensor design literature^27^.

**Fig. 11:**
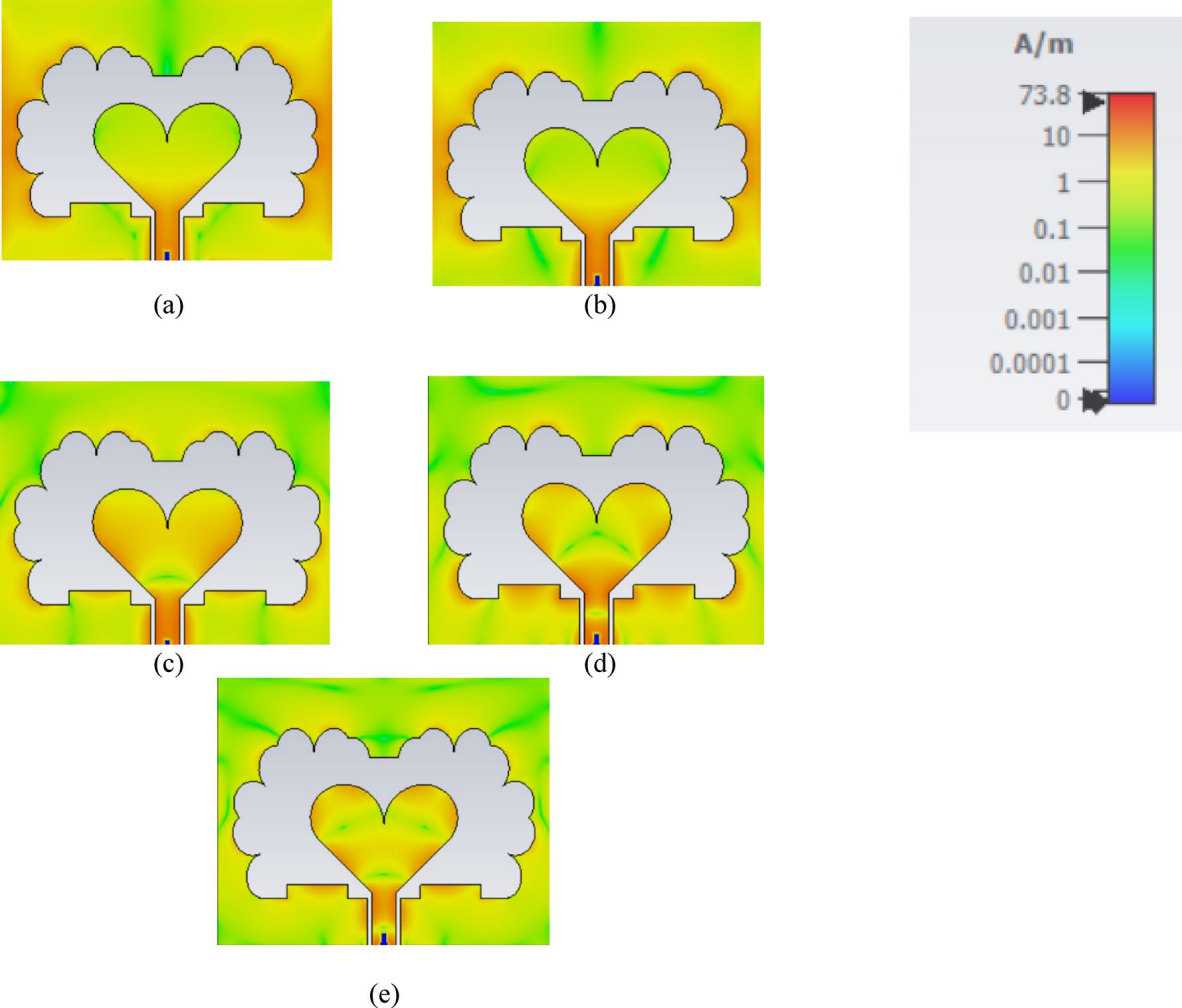
Surface current density distribution of the proposed sensor at different frequencies: (**a**) 2 GHz, (**b**) 3 GHz, (**c**) 10 GHz, (**d**) 15 GHz, (**e**) 17 GHz.

The surface current distribution, illustrated in Fig. 11(a–e), was analyzed at 2 GHz, 3 GHz, 10 GHz, 15 GHz, and 17 GHz to investigate the sensor’s electromagnetic behavior across its operational range. At 2 GHz and 3 GHz, the surface currents are primarily concentrated around the heart-shaped tuning stub and along the edges of the heart-shaped slots in the DGS, indicating effective excitation of the fundamental resonant modes^29^. At 10 GHz, the current begins to spread more extensively over the ground plane, especially near the periphery of the DGS, reflecting the excitation of higher-order modes. At 15 GHz, the current density increases significantly and becomes more localized at the lower part of the tuning stub and slot edges, indicating strong coupling and enhanced radiation efficiency. At 17 GHz, a denser and more uniform distribution appears across both the stub and the surrounding ground, confirming the sensor capability to maintain strong resonance and efficient radiation even at higher frequencies.

### Durability study of the proposed sensor

To assess the robustness of the proposed UWB CPW sensor under wearable conditions, the effect of mechanical deformation was investigated through full-wave simulations. Two common deformation modes were considered: bending and crumpling. Bending was chosen because the human chest is naturally curved rather than flat, and thus the sensor must conform to this shape during practical use^34^. Crumpling was included since the body is not stable during daily activities and sleeping; involuntary movements often cause local folding or wrinkling of the sensor surface^19^. The analysis focused on the reflection coefficient (S11) as the primary performance indicator, since it directly reflects impedance matching and operational bandwidth stability^35^.

### Bending

#### Simulation setup

**Fig. 12:**
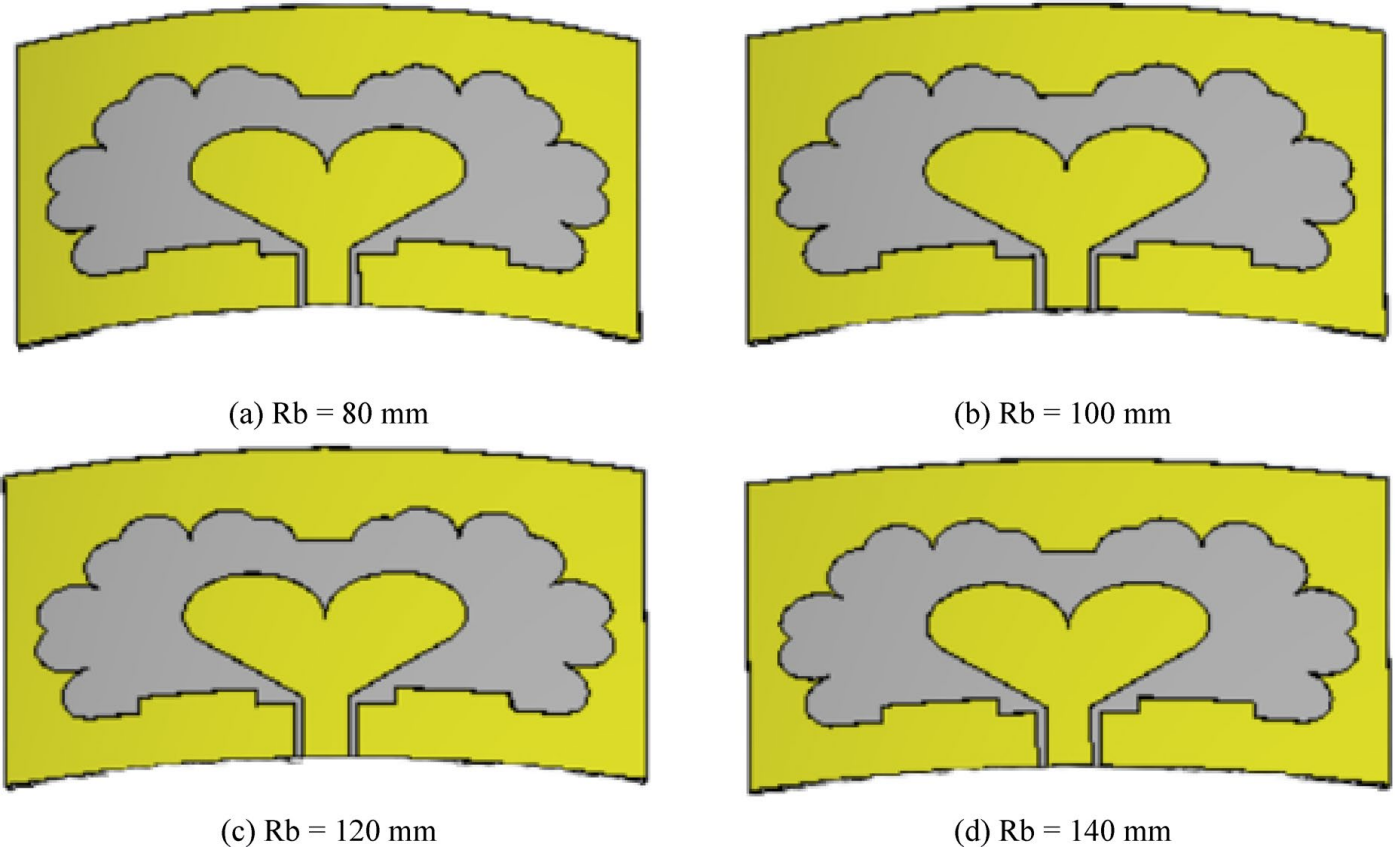
Depiction of the bent sensor at radii (**a**) 80 mm, (**b**) 100 mm, (**c**) 120 mm and (**d**)140 mm. (**c**) Rb = 120 mm, (d) Rb = 140 mm.

Bending was simulated by wrapping the sensor around cylindrical surfaces of radii 80, 100, 120, and 140 mm, as illustrated in Fig. 12(a–d). The metallization layer was placed on the outer side of the curvature, and deformation was applied along the sensor’s longitudinal axis. The bending radii 80, 100, 120, and 140 mm were selected to represent realistic chest curvatures across different human groups. Smaller radii such as 80 mm mimic the chest curvature of younger or smaller-sized individuals with narrower torsos, while larger radii like 120–140 mm reflect the broader and flatter chest profiles often seen in adults and elderly individuals^36^. This range ensures that the sensor performance is tested under bending conditions representative of real anatomical variations, covering both tighter and more relaxed chest curvatures likely to be encountered in practical biomedical monitoring applications.

#### Simulation results

**Fig. 13:**
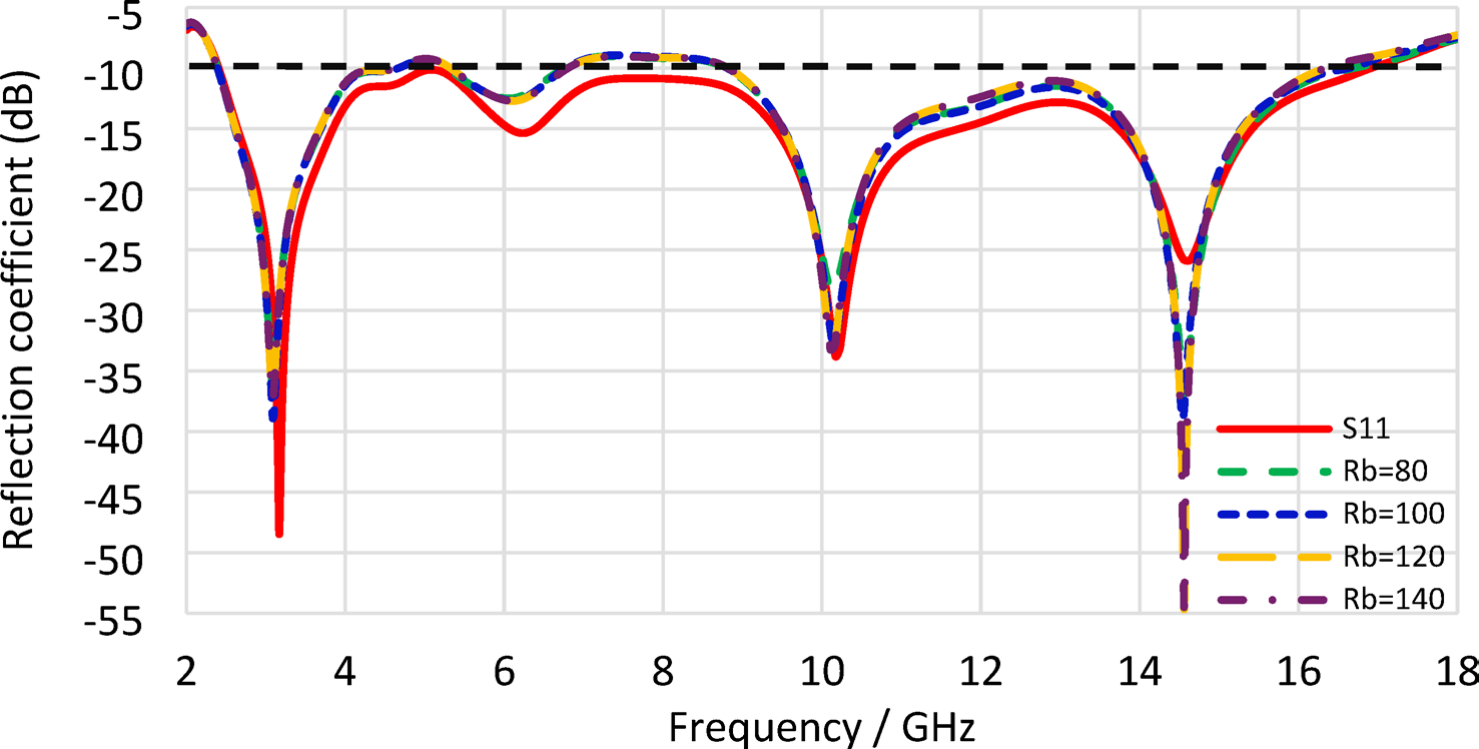
S11| versus frequency for flat and all bending cases at radii 80 mm, 100 mm, 120 mm and 140 mm.

Figure 13 illustrates the simulated S11 curves of the proposed sensor under different bending radii (Rb = 80, 100, 120, and 140 mm) compared to the flat condition. Across all cases, the sensor preserved wideband impedance matching within the 2.4–17 GHz range, validating its suitability for UWB applications. As the bending radius decreased, only slight frequency shifts and variations in resonance depth were observed. The most noticeable effect occurred at Rb = 80 mm, which represents tighter chest curvatures, where a small upward frequency shift and reduced resonance depth can be seen. Nevertheless, the reflection coefficient remained consistently below − 10 dB across the band, confirming that the CPW slot geometry offers strong robustness against bending-induced detuning.**Figure.13.** |S11| versus frequency for flat and all bending cases at radii 80 mm, 100 mm, 120 mm and 140 mm.

#### Measurement results

**Fig. 14:**
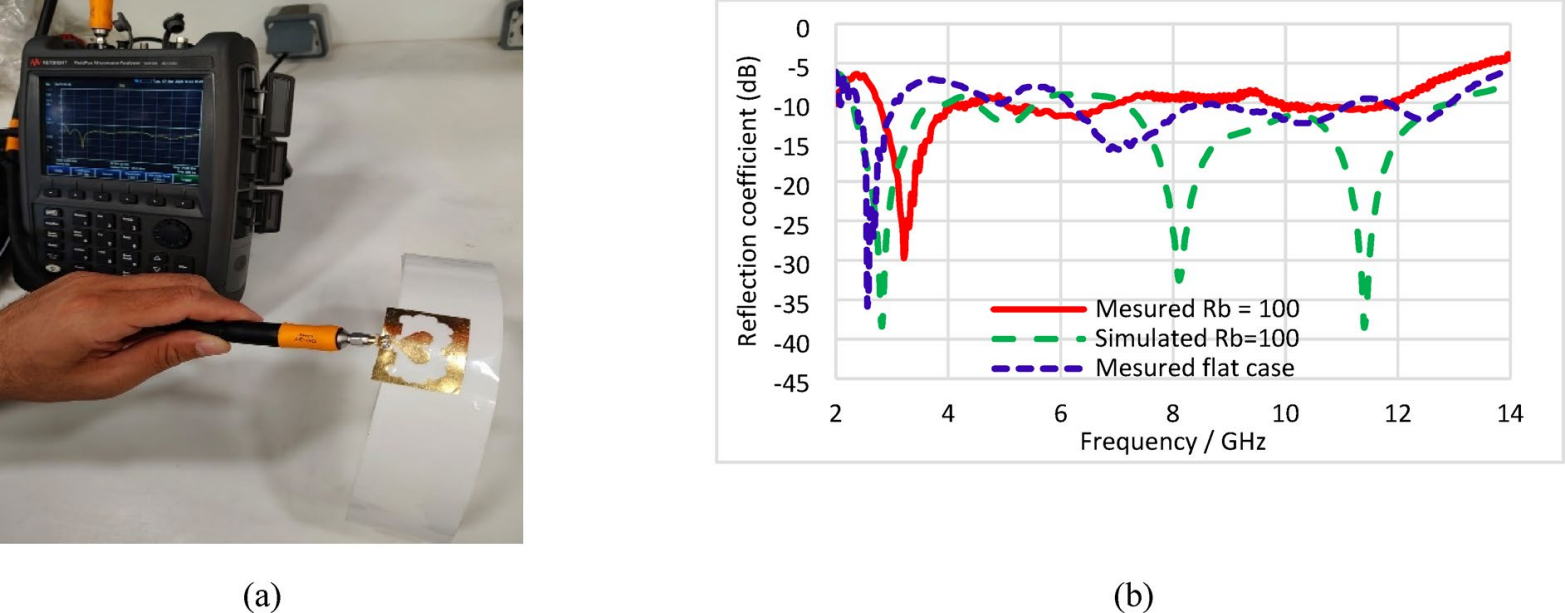
(**a**) Measurement setup of bending case Rb = 100 mm, (**b**) Measured and simulated bending case Rb = 100 mm.

To experimentally validate the sensor performance under deformation, the fabricated prototype was measured using the setup shown in Fig. 14(a) The reflection coefficient (S11) was recorded using a vector network analyzer under both flat and bent conditions. Figure 14(b) shows the measured S11 of the sensor bent with a bending radius of Rb = 100 mm, compared with the measured flat-case result and the corresponding simulated bent-case result. It can be observed that the measured bent response follows the same general trend as the simulated one, confirming the reliability of the simulation analysis. Some discrepancies and frequency shifts are noticed due to fabrication tolerances, manual bending imperfections, and measurement uncertainties. In comparison with the flat-case measurement, the bent sensor maintains acceptable impedance matching over the operating band, indicating that the proposed sensor is robust under practical bending conditions and suitable for wearable applications.

### Crumpling

#### Simulation setup

**Fig. 15:**
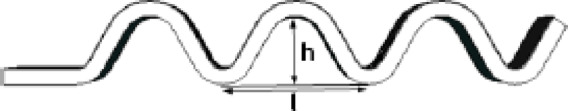
Depiction of the crumpling parameter^36^.

**Fig. 16:**
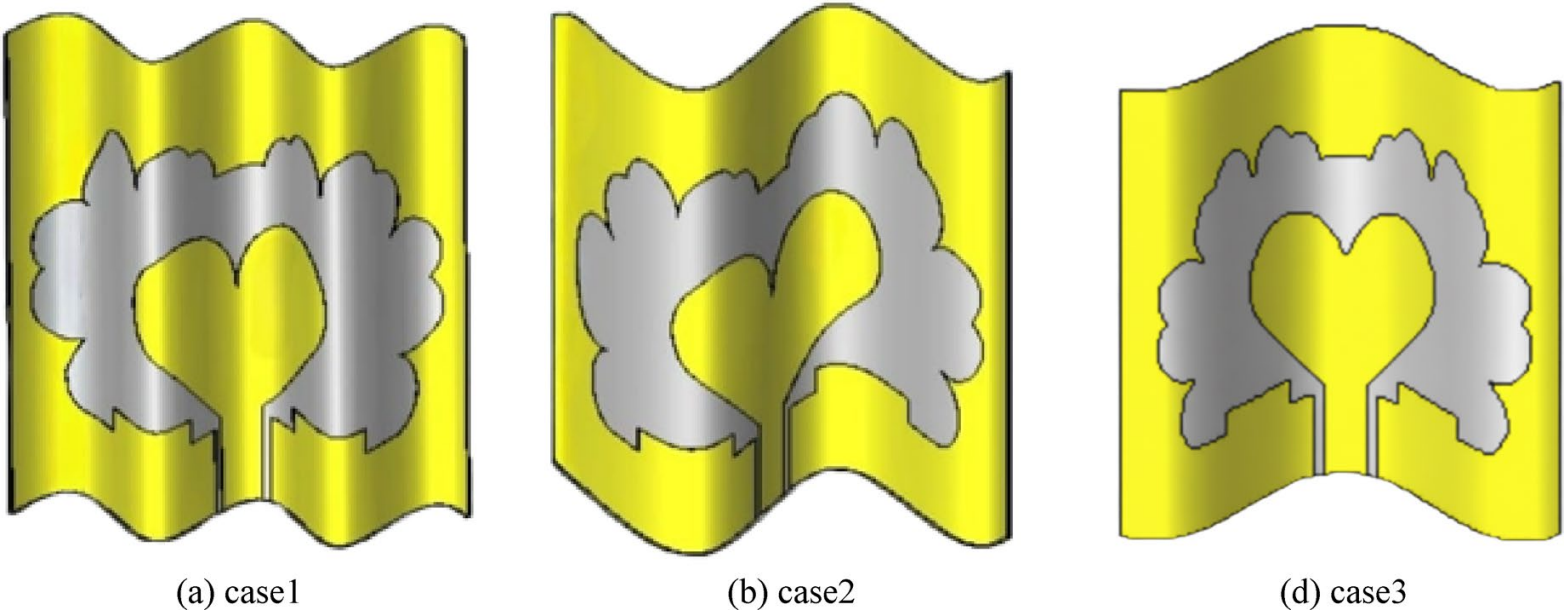
Depiction of the crumpled sensor at different cases (**a**) case1, (**b**) case2 and (**c**) case3.

Crumpling was simulated by introducing horizontal wave-like deformations across the entire 50 × 40 mm² sensor surface. The deformation was modeled as periodic folds defined by a crumple height (h) and crumple length (l) as shown in Fig. 15, representing the vertical depth and horizontal spacing of the wrinkles, respectively. Three cases as illustrated in Fig. 16(a–c) were considered to reflect different real-life severities: mild crumpling (h = 8 mm, l = 6 mm), moderate crumpling (h = 11 mm, l = 10 mm), and severe crumpling (h = 15 mm, l = 14 mm). These dimensions were chosen to realistically mimic wrinkles and folds that occur during daily body movements and while sleeping, when the sensor is in continuous contact with the human torso. Smaller crumpling lengths and larger heights (severe case) replicate tight fabric-like folds formed under body pressure, while longer lengths with shallow heights (mild case) reflect softer wrinkles caused by relaxed motion. This range ensures that the sensor performance is evaluated under deformation conditions likely to be encountered in practical biomedical monitoring applications, particularly in scenarios such as epilepsy detection during sleep^36^.

#### Simulation results

**Fig. 17:**
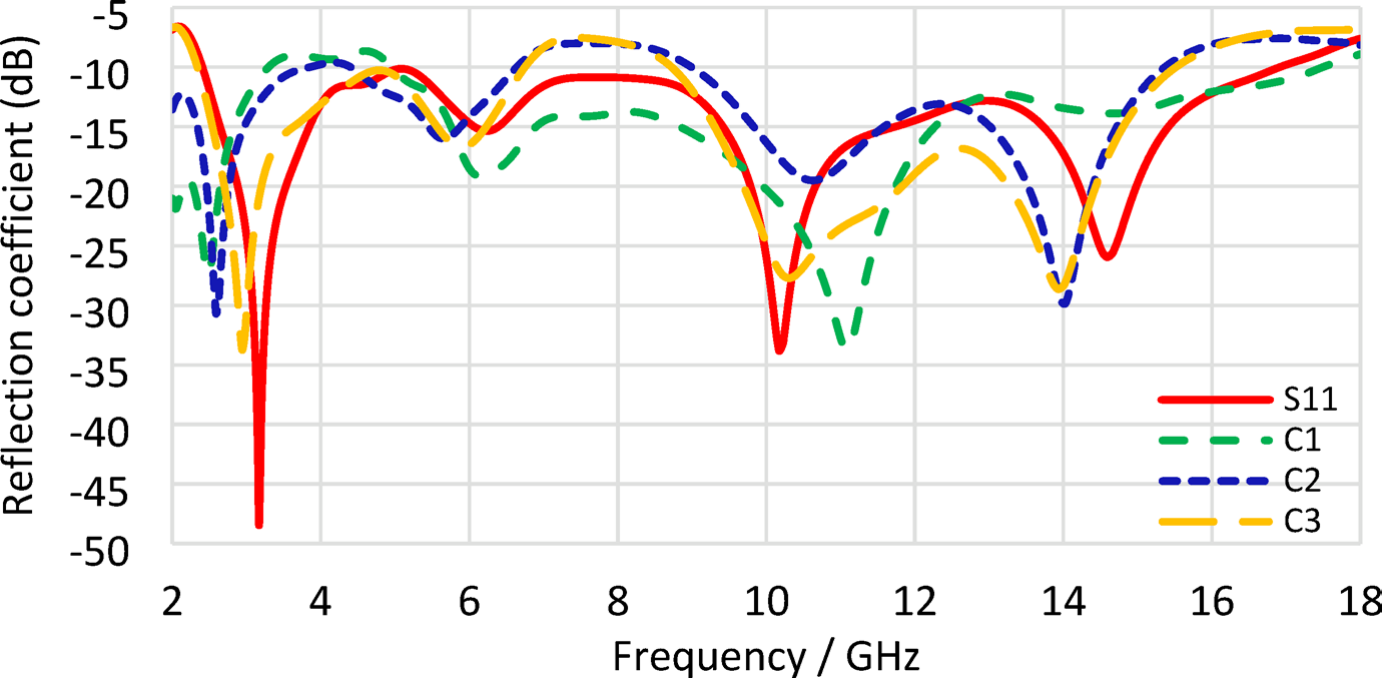
S11| versus frequency for flat and all crumpling cases (C1, C2 and C3).

Figure 17 presents the simulated S11 response of the sensor under three crumpling conditions (C1, C2, and C3) compared with the flat state. In all cases, the sensor sustained a wide impedance bandwidth from 2.8 to 17 GHz, confirming its suitability for UWB applications. Crumpling introduced minor frequency shifts and variations in resonance depth, most evident in the 2.8–4 GHz range, where the deep notch of the flat case (− 45 dB) became shallower (− 20 to − 30 dB). In the mid-to-high frequency bands (≈ 6–15 GHz), the resonances were largely preserved. While moderate crumpling (C2) slightly enhanced the resonance at ~ 14 GHz, the flat case exhibited the deepest notch around 10 GHz, with all crumpled cases showing shallower responses. Severe crumpling (C3) improved the resonance near 6 GHz but caused modest degradation in the upper bands. Overall, the sensor consistently maintained reflection coefficient below − 10 dB across the band, demonstrating strong resilience to crumpling-induced deformation. Although minor degradation in the reflection coefficient is observed in some frequency regions under crumpling conditions, including localized values approaching − 10 dB, this impedance mismatch does not significantly affect the overall sensing performance. In the proposed system, the primary sensing mechanism is based on the phase variation of the transmission coefficient (S21) between the transmitting and receiving sensors rather than the absolute magnitude of S11. Therefore, slight variations in input matching mainly influence radiation efficiency and received power level, while the phase stability required for respiration monitoring remains largely preserved. Moreover, the sensor maintains a wide operational bandwidth, ensuring sufficient signal coupling between both sensors even under deformation conditions, which supports reliable breathing rate estimation in practical wearable scenarios.

#### Measurement results

**Fig. 18:**
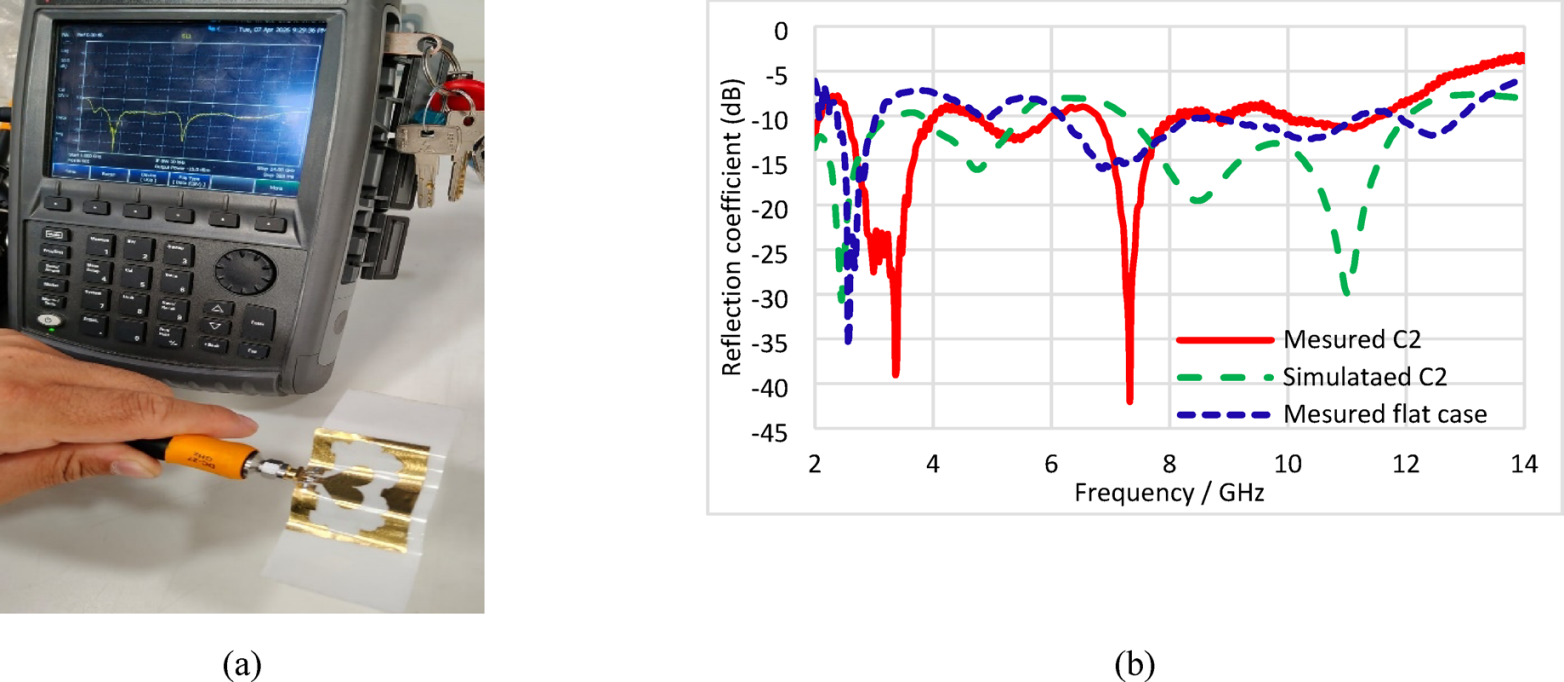
(**a**) Measurement setup of crumpling case 2 (C2), (**b**) Measured and simulated crumpling case 2(C2).

To further investigate the effect of severe deformation, the fabricated sensor was experimentally measured under the crumpling condition using the measurement setup shown in Fig. 18(a). The reflection coefficient (S11) was recorded using a vector network analyzer and compared with both the measured flat-case result and the corresponding simulated crumpled-case result, as illustrated in Fig. 18(b). The measured result for Crumpling Case 2 (C2) shows a similar overall trend to the simulated response, with noticeable shifts in resonant frequencies and variations in matching levels. These differences can be attributed to fabrication tolerances, the non-uniform nature of manual crumpling, and measurement uncertainties. Nevertheless, the sensor preserves acceptable impedance matching across a wide frequency range compared with the flat-case measurement, demonstrating that the proposed design maintains stable performance even under severe deformation, which is desirable for wearable biomedical applications.

### SAR analysis

**Fig. 19:**
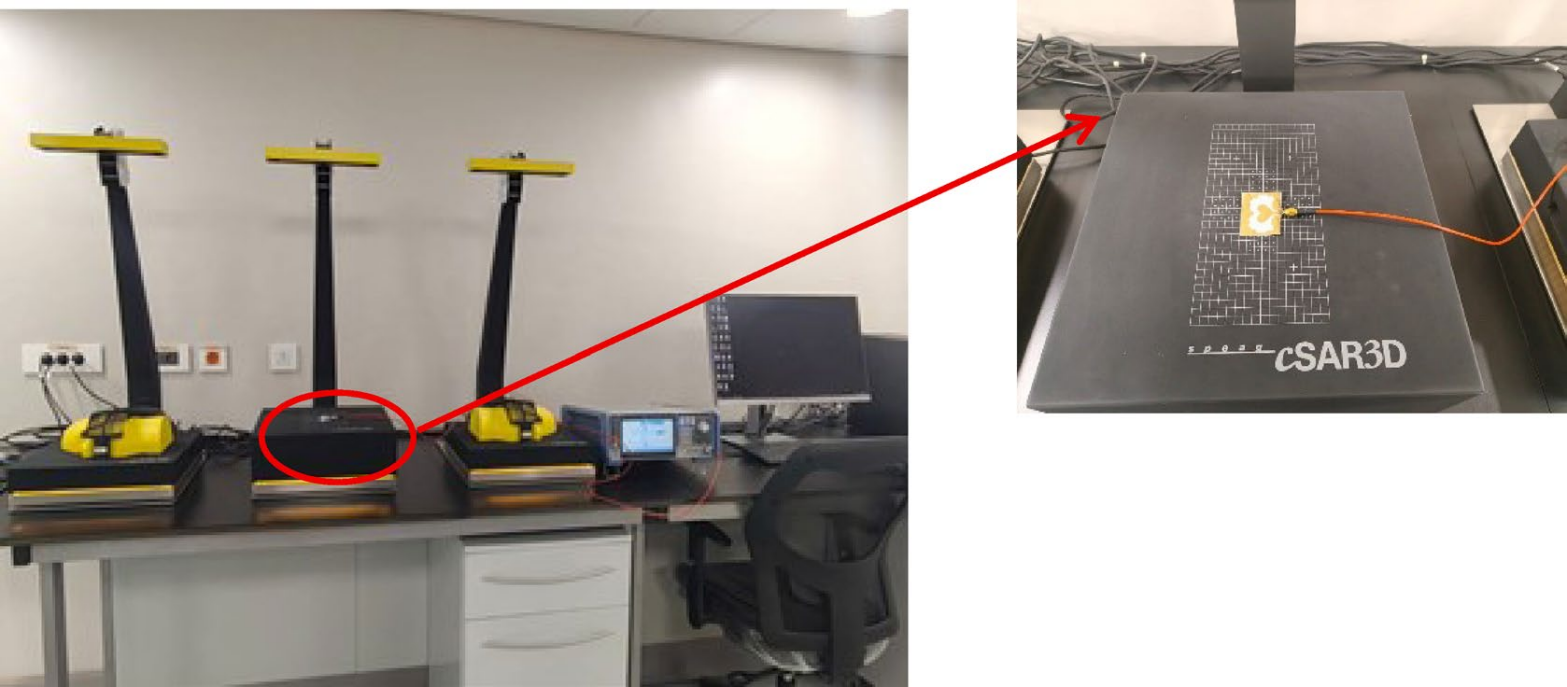
SAR measurement setup.

The SAR for the proposed UWB CPW sensor was evaluated experimentally using a standardized phantom-based measurement system (R&S CMX500 OBT with SPEAG DASY8) which is based on tissue-equivalent phantom materials to emulate the dielectric properties of human tissues. The measurement setup, as depicted in Fig. 19, involves placing the sensor in direct contact with the phantom to accurately represent the exposure conditions when worn on the human chest^36^. The SAR is determined based on the measured electric field distribution within the phantom and evaluated according to international safety standards, particularly the IEEE C95.3 standard, which specifies maximum localized SAR limits of 1.6 W/kg averaged over 1 g of tissue and 2.0 W/kg averaged over 10 g of tissue^37^. Compliance with these limits ensures safe operation for continuous biomedical monitoring applications. Measurements were conducted as shown in Table 2 at three representative frequencies within the UWB range (3 GHz, 4.5 GHz, and 6 GHz) and at three transmitted power levels: 10 dBm, 15 dBm, and 20 dBm. The measured SAR values (SAR(1 g) and SAR(10 g)) show an increasing trend with both frequency and transmitted power, which is consistent with electromagnetic absorption characteristics in biological tissues. Even at the highest tested power of 20 dBm (100 mW) and frequency of 6 GHz, the maximum SAR(1 g) is 0.946 W/kg and SAR(10 g) is 0.258 W/kg, both remaining well below the safety limits. These results confirm the electromagnetic safety of the proposed sensor for continuous wearable operation. In practical breathing monitoring scenarios, the required transmission power is expected to be significantly lower, providing an additional safety margin^36^.

**Fig. 20:**
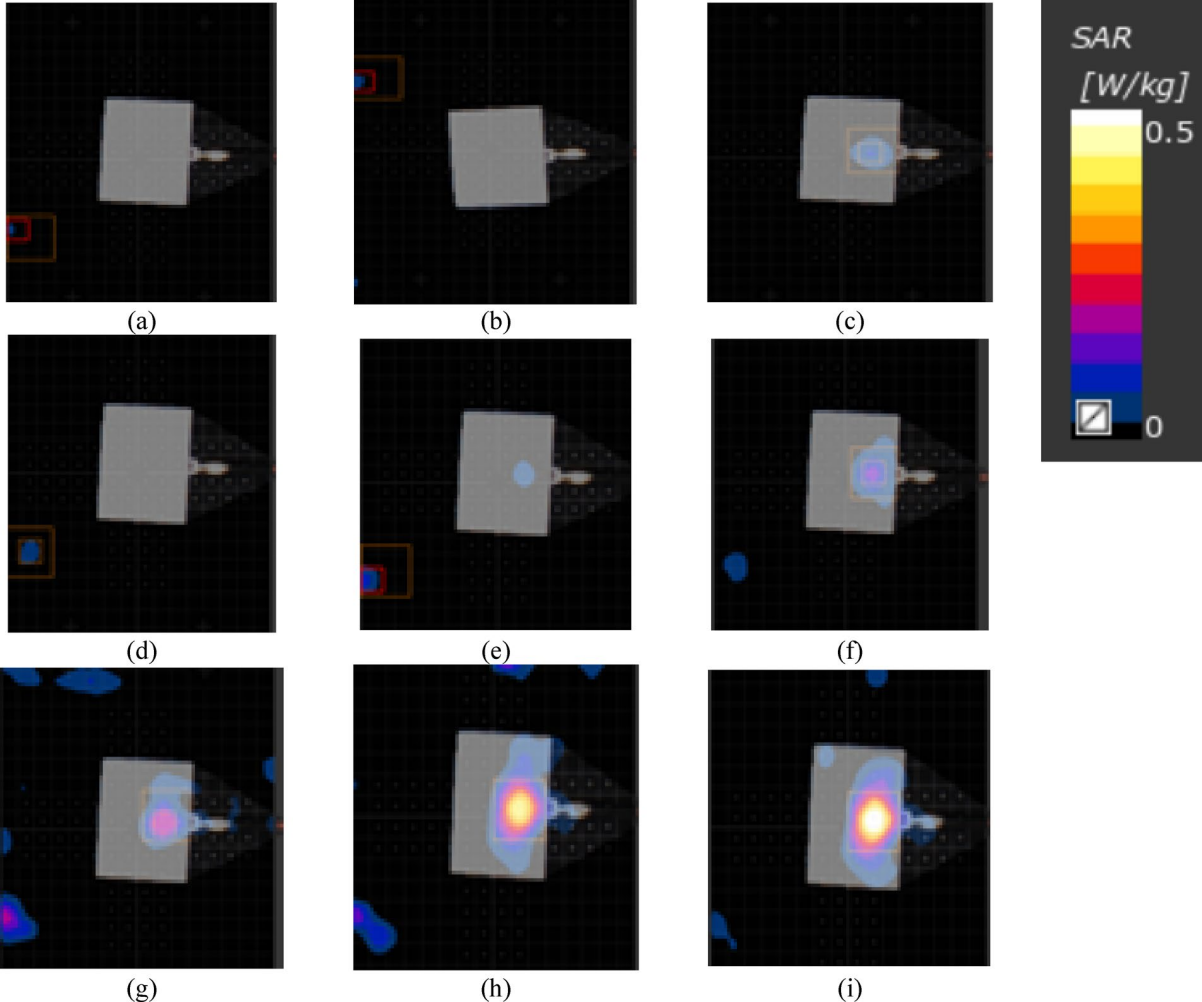
SAR measurements of the proposed sensor (**a**) SAR at 3 GHz and 10 dB, (**b**) SAR at 3 GHz and 15 dB, (**c**) SAR at 3 GHz and 20 dB, (**d**) SAR at 4.5 GHz and 10 dB, (**e**) SAR at 4.5 GHz and 15 dB, (**f**) SAR at 4.5 GHz and 20 dB, (**g**) SAR at 6 GHz and 10 dB, (**h**) SAR at 6 GHz and 15 dB, (**i**) SAR at 6 GHz and 20 dB.

**Table 3:** Comparing for the suggested wearable sensor.

Frequency	Power level	Sar(1g)	Sar(10g)
3GHz	10dB	0.022	0.005
	15dB	0.031	0.009
	20dB	0.059	0.020
4.5GHz	10dB	0.036	0.008
	15dB	0.045	0.008
	20dB	0.090	0.028
6GHz	10dB	0.150	0.038
	15dB	0.296	0.081
	20dB	0.946	0.258

### Proposed respiratory monitoring system with potential epilepsy applications

Epileptic seizures are frequently accompanied by distinct physiological changes, and among these, alterations in respiratory rhythm are especially significant^5^. Continuous monitoring of breathing patterns can therefore serve as a potential non-invasive indicator for epilepsy-related monitoring^7^. In this work, the same flexible UWB CPW-based respiratory sensing system described earlier is adapted for epileptic monitoring applications^10^. By analyzing the variations in the transmission coefficient phase (S₂₁), the system can detect irregularities in breathing that may be associated with pre-seizure physiological changes^8^. This approach enables continuous and comfortable monitoring suitable for both clinical and home settings.

### Principle of operation

**Fig. 21:**
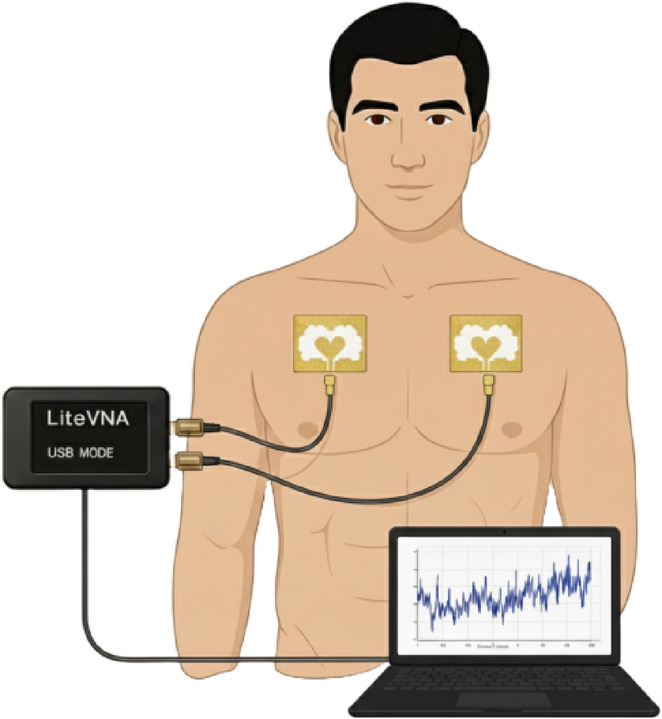
The proposed system.

The proposed wearable system continuously monitors respiratory activity using a flexible RF sensing configuration^10^. The system adopts an established RF-based vital sign monitoring technique reported in the literature, where respiratory motion is extracted using variations in the transmission coefficient (S₂₁) between a transmitter–receiver sensor pair placed on the chest^8^. Mechanical expansion and contraction of the chest cause subtle changes in the S₂₁ phase, which are tracked over time to extract the respiratory pattern^9,17^. The system operates by converting mechanical chest motion into phase variations of RF signals, ensuring robustness against external electromagnetic interference. The overall configuration, illustrated in Fig. 21, includes two identical sensors acting as transmitting (Tx) and receiving (Rx) sensors, a Nano Vector Network Analyzer (Nano-VNA) serving as the transceiver, and a laptop for data acquisition and processing. The Tx sensor emits a low-power RF signal toward the chest, while the Rx sensor receives the transmitted signal after interaction with body tissues. The lite VNA records both signals, allowing precise extraction of the phase information correlated with breathing^17^.

When normal breathing occurs, the amplitude and phase of the received signal vary in a quasi-sinusoidal manner, corresponding to the inhalation and exhalation cycles^15^. However, during breath-holding or shallow breathing, the phase fluctuation magnitude decreases. This dynamic enables the system to distinguish between different respiratory states. By continuously monitoring S21 phase variations, the breathing rate can be extracted through signal demodulation and spectral analysis techniques such as Fast Fourier transform (FFT) or autocorrelation methods. The system operation relies on the high temporal resolution and wide bandwidth of UWB signals, which allow the detection of displacement changes on the order of millimeters caused by chest motion. Unlike optical or mechanical sensors, this RF-based sensing approach operates through direct contact with the chest while maintaining flexibility and reduced sensitivity to motion artifacts, offering high accuracy even under slight body movement^11^. The transparent PVC substrate and gold-leaf conductive layer ensure conformal skin attachment, minimizing air gaps and improving coupling efficiency between the sensors and the chest surface^33^. For potential epilepsy-related applications, the principle is based on the observation that abnormal respiratory patterns often precede seizure onset^5^. A sudden increase or irregularity in the breathing rate, identified through deviations in the extracted phase pattern, may serve as a potential early indicator of abnormal respiratory behavior associated with seizures^7^. The system can be integrated with a microcontroller or wireless transceiver to process the signal in real time and trigger alerts when respiratory parameters exceed a predefined threshold^4^. Compared to conventional UWB sensor fabrication techniques such as inkjet printing, screen printing, and photolithography, which require expensive equipment, conductive inks, cleanroom facilities, and post-processing steps, the proposed design offers a low-cost and simplified fabrication approach^32^. It relies only on commercially available materials and basic fabrication tools, eliminating the need for specialized manufacturing infrastructure while maintaining suitable performance for wearable biomedical applications.

### Determination of optimal sensors location

The performance of the proposed RF-based respiratory monitoring system is highly dependent on the stability of the wireless link and the sensitivity of the S21 phase to changes in the chest wall’s surface distance^8^. To determine the optimal placement configuration for maximizing signal fidelity and robustness, a comparative study was performed by varying the separation distance and orientation between the Tx and Rx sensors on the subject’s torso.

All procedures involving human participants were conducted in accordance with relevant institutional guidelines, national regulations, and ethical standards. The study protocol was reviewed and approved by the Research Ethics Committee of the Faculty of Engineering at Shoubra, Benha University (Research Code: ELE 24/25–46, approval date: 11 November 2025). The experimental measurements were performed on an adult healthy volunteer using non-invasive wearable UWB sensor placed on the chest in different positions for respiratory rate monitoring. Informed consent was obtained from the participant prior to participation in the study.

**Fig. 22:**
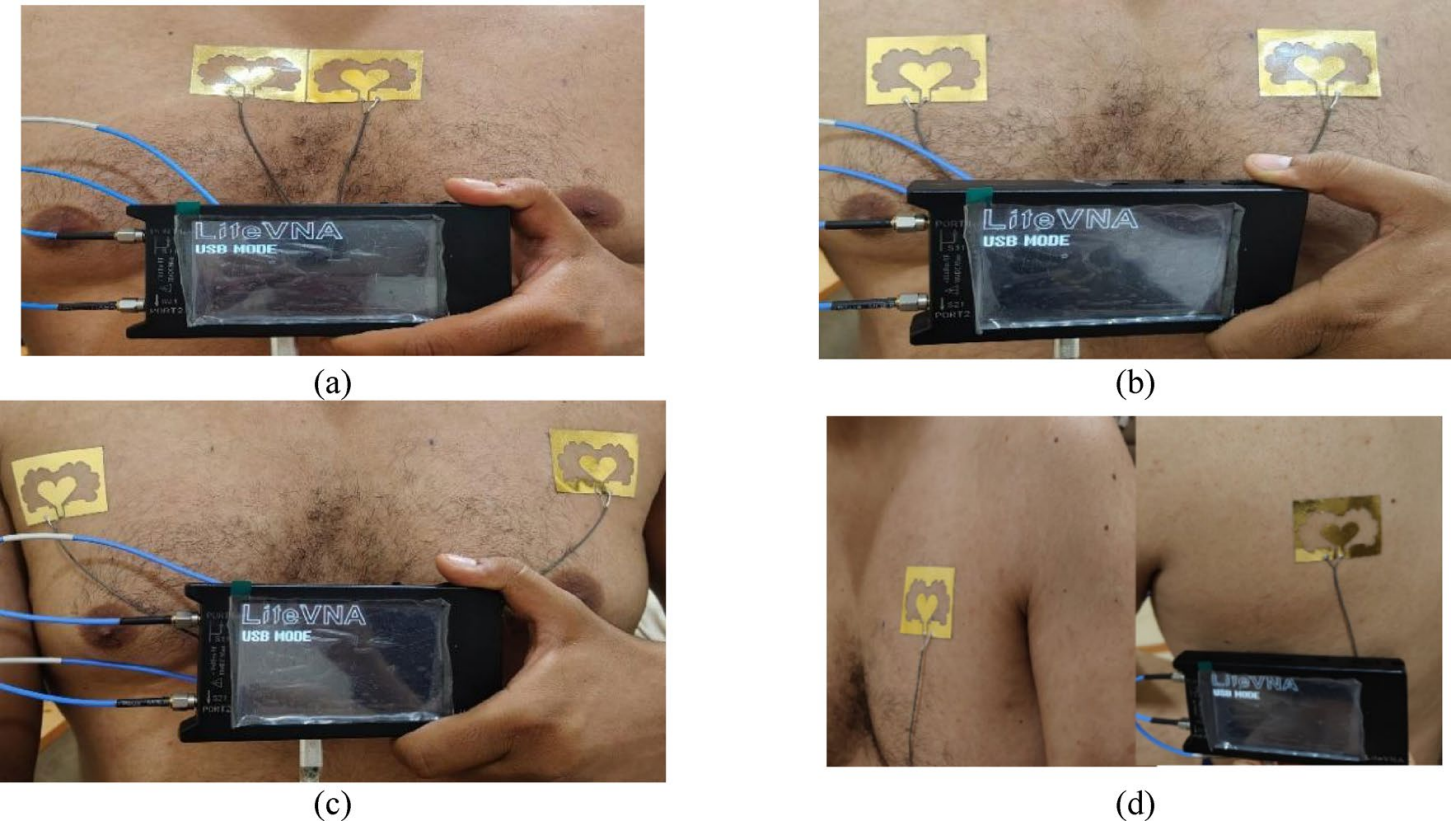
Experiment setup for Breathing rate with different configurations (**a**) 0 cm separation, (**b**) 10 cm separation, (**c**) 20 cm separation and (**d**) Front-to-Back.

The proposed S21-based respiratory monitoring system can be influenced by both environmental and human-related factors that affect measurement stability and accuracy. Environmental factors include the presence of nearby reflecting objects such as walls, furniture, and surrounding electronic devices, which may introduce variations in the measured phase response due to multipath effects and electromagnetic coupling from ambient sources. Human-related factors include variations in sensor placement on the chest, differences in subject posture during measurement, and involuntary body movements other than respiration. To mitigate these effects, all measurements are conducted in a consistent real-world environment where the same physical location is used for all recordings, and surrounding objects are kept unchanged throughout the measurement sessions. Additionally, sensor placement is carefully standardized on the chest, and subjects are instructed to maintain a stable posture and minimize unnecessary movement during data acquisition to ensure that the observed S21 variations are primarily attributed to respiratory activity. The four tested configurations involved placing both sensors on the chest with varying horizontal separations (0 cm, 10 cm, and 20 cm), and a final configuration with the Tx sensor on the anterior chest and the Rx sensor on the posterior back, as shown in Fig. 22. The raw S21 phase variation, directly correlated with breathing cycles, was recorded for each setup over a 60-second period.

#### Separation of 0 cm (adjacent placement)

**Fig. 23:**
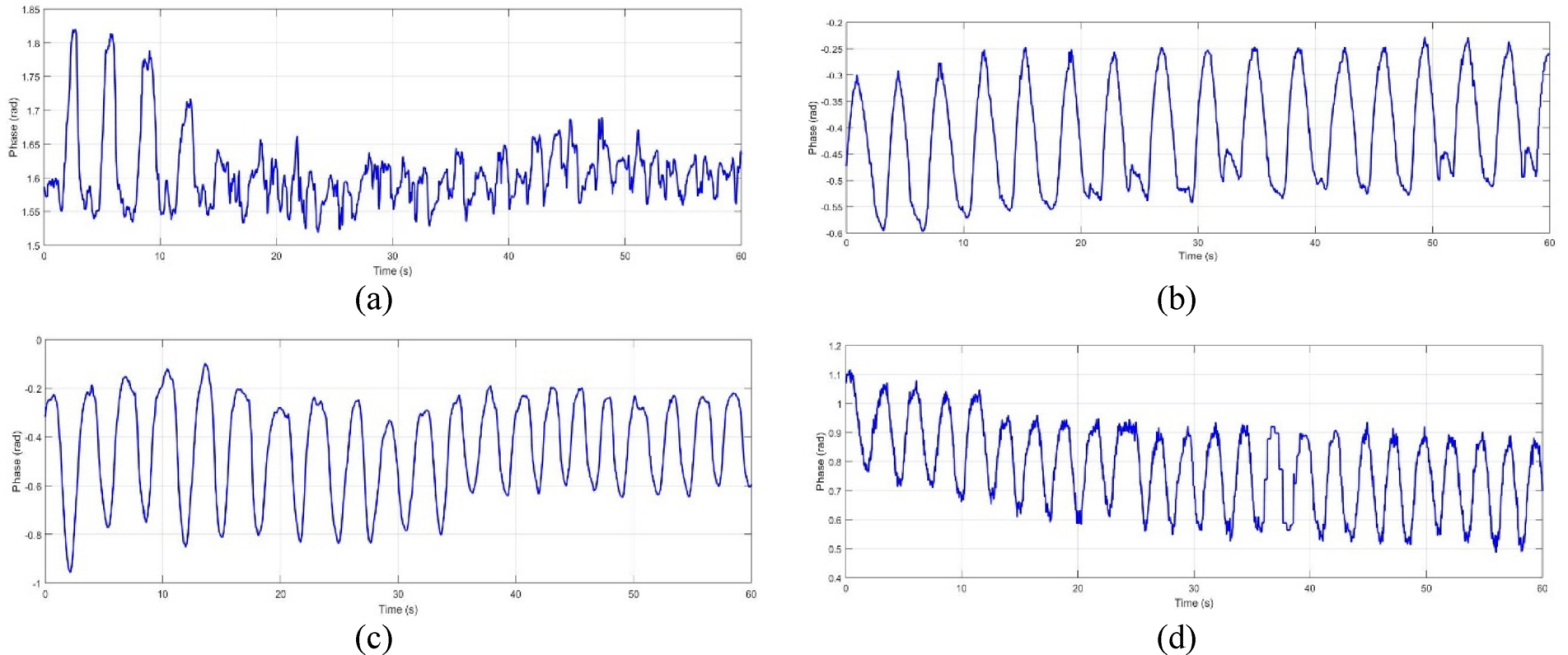
Breathing rate with different configurations (**a**) 0 cm separation, (**b**) 10 cm separation, (**c**) 20 cm separation and (**d**) Front-to-Back.

In this configuration, the sensors were placed immediately adjacent to each other. As shown in Fig. 23(a), the recorded phase signal exhibited significant noise and instability. The respiratory pattern is visible but is severely masked by a high level of distortion and signal magnitude fluctuations, particularly between the 10-second and 40-second marks. This poor performance is attributed to the strong direct coupling between the two sensors when they are in direct contact. The proximity maximizes the electromagnetic energy traveling directly between the sensors (the line-of-sight path) over the signal path modulated by chest movement, resulting in a low signal-to-noise ratio (SNR) for the physiological signal of interest. The respiratory signal amplitude (peak-to-peak phase difference) is minimal and poorly defined in this case, making reliable breathing rate extraction challenging.

#### Separation of 10 cm

Increasing the separation to 10 cm significantly improved the signal quality. As evidenced by Fig. 23(b), this configuration yielded a clean, stable, and highly sinusoidal phase response over the entire 60-second duration. The signal amplitude is clearly defined, with a consistent peak-to-peak phase variation of approximately $\:0.4{\:}^{o}$. This separation is large enough to substantially reduce the direct coupling path, forcing the signal to travel a path that is more sensitive to the millimeter-scale changes in the chest’s curvature and distance. The enhanced periodicity and magnitude stability confirm this 10 cm separation as a highly efficient configuration for respiratory sensing.

#### Separation of 20 cm

When the sensors were separated by 20 cm, the signal quality deteriorated slightly compared to the 10 cm case Fig. 23(c). Although the sinusoidal respiratory rhythm is still clearly distinguishable, the overall signal amplitude is visibly reduced, and minor noise components are re-introduced, particularly after the 30-second mark. While still viable, the increased distance likely reduces the overall received signal strength (RSS) and makes the system more susceptible to slight movement artifacts or internal body scattering losses^19^.

#### Front-to-back (Transmissive) placement

The final test utilized a transmissive arrangement, placing the Tx sensor on the anterior chest and the Rx sensor on the posterior back Fig. 23(d). This configuration requires the signal to propagate entirely through the body tissue^19^. The resulting S21 phase data showed a distinct respiratory pattern, confirming the feasibility of through-body sensing. However, the signal exhibited a pronounced baseline drift and a lower overall magnitude stability compared to the 10 cm coplanar case. Propagation through the human torso introduces significant attenuation and dispersion, impacting the phase stability and signal strength. Furthermore, the signal magnitude is likely influenced not only by chest expansion but also by internal physiological processes and organ movement^13^. Based on the experimental data, the configuration with10 cm separation on the anterior chest provides the optimal balance between high signal-to-noise ratio (by mitigating direct coupling) and sufficient sensitivity to chest wall displacement. This coplanar arrangement minimizes signal attenuation from deep-body transmission while providing a clean, high-amplitude, and stable phase response essential for reliable, continuous respiratory rate extraction in clinical and home environments. The10 cm separation will therefore be adopted as the default placement for subsequent development of epilepsy-related monitoring applications.

### Validation on multiple participants and respiratory signal analysis

**Fig. 24:**
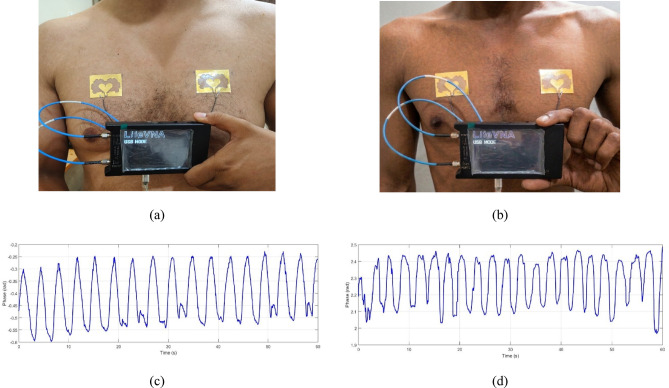
(**a**) Experimental setup for the first participant (29 years old), (**b**) Experimental setup for the second participant (21 years old), (**c**) Recorded S21 phase variation signal of the first participant under resting conditions, (**d**) Recorded S21 phase variation signal of the second participant under resting conditions.

To further validate the repeatability and reliability of the proposed respiratory monitoring system, additional measurements were conducted on a second healthy volunteer using the optimal 10 cm separation identified in the previous subsection. The experimental setup for the first and second participants is shown in Fig. 24(a) and Fig. 24(b), respectively. The recorded S21 phase variation signals for both participants under normal resting conditions are presented in Fig. 24(c) and Fig. 24(d). In both cases, clear periodic oscillations corresponding to inhalation and exhalation cycles can be observed, confirming the capability of the proposed system to monitor respiratory activity across different individuals. In addition to variations in signal amplitude and waveform shape, a difference in the number of detected breathing cycles (respiratory rate) is also observed between the two participants, which is expected due to natural physiological variability among individuals. These differences are attributed to variations in breathing patterns, chest morphology, and slight differences in sensor placement. Overall, these results demonstrate the repeatability and robustness of the proposed wearable UWB CPW sensing system for continuous respiratory monitoring.

**Fig. 25:**
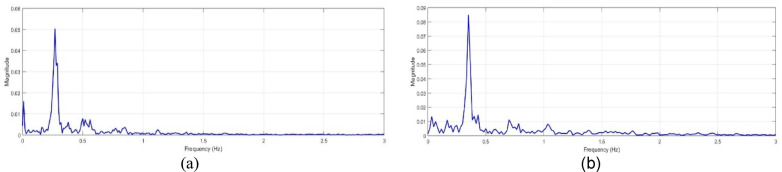
(**a**) FFT of the first participant showing a dominant frequency of 0.27 Hz (16 BPM), and (**b**) FFT of the second participant showing a dominant frequency of 0.35 Hz (21 BPM).

To further analyze the respiratory signals detected by the proposed sensor, the phase variation of the S21 parameter was processed using Fast Fourier Transform (FFT). This analysis enables identification of the dominant frequency component of respiration, providing a clear estimation of the breathing rate. The FFT analysis of the recorded phase signals shown in Fig. 25 revealed dominant frequencies of 0.27 Hz and 0.35 Hz for the first and second participants, respectively. These frequencies correspond to equivalent respiration rates of approximately 16 BPM and 21 BPM. The variation in respiratory rate between the two participants is expected due to natural physiological differences. These results confirm the accuracy and reliability of the proposed sensor in capturing and quantifying respiratory cycles.

### Sensitivity of the proposed system to rapid respiratory variations

**Fig. 26:**
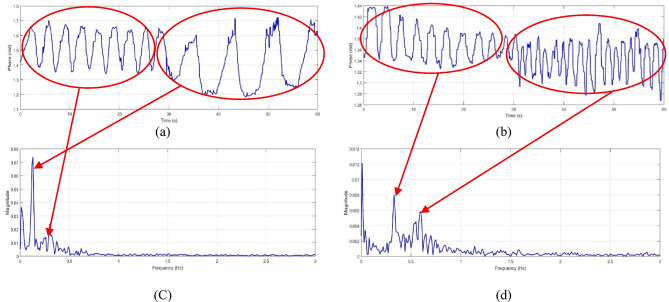
(**a**) Time-domain signal of a slow respiratory disturbance. (**b**) Time-domain signal of a fast respiratory disturbance. (**c**) FFT of the slow disturbance showing a dominant peak at 0.13 Hz. (**d**) FFT of the fast disturbance showing a dominant peak at 0.57 Hz.

The potential clinical relevance of the proposed UWB CPW sensor for epilepsy-related monitoring is based on its high sensitivity to periictal respiratory abnormalities. As established in clinical literature, seizures are frequently preceded or accompanied by significant deviations in breathing rhythm, such as tachypnea (rapid breathing) or bradypnea (slow breathing), resulting from autonomic dysregulation and abnormal cortical discharges. The proposed system demonstrates a robust capability to detect these transitions with high precision through the analysis of S21 phase variations. To validate the accuracy and reliability of the sensor, controlled respiratory simulations were performed by intentionally accelerating and decelerating the breathing rate, emulating tachypneic and bradypneic patterns. All experiments were therefore conducted at a fixed TX–RX separation of 10 cm, as previously identified in the preceding subsection as the distance yielding the most reliable respiratory measurement. During stable resting periods or postictal recovery phases, breathing often manifests as slow, rhythmic cycles. The time-domain results for slow respiration as seen in Fig. 26(a) show clear, high-amplitude periodic oscillations. This confirms that the sensor accurately tracks the full mechanical displacement of the chest wall during expansion and contraction, producing stable, periodic phase fluctuations.

The spectral analysis FFT of this phase signal as seen in Fig. 26(c) reveals a sharp, dominant magnitude peak at approximately 0.13 Hz. This frequency corresponds to a slow respiratory rate of 8 BPM, providing a high-fidelity baseline for the subject. The narrowness and clarity of the peak indicate a high signal-to-noise ratio (SNR), which is essential for identifying regular breathing cycles amidst minor bodily movements. A critical feature for early seizure warning is the detection of tachypnea, where the respiratory rate exceeds normal resting values deviation that can begin seconds to minutes before clinical seizure onset. As shown in the fast breathing time-domain results Fig. 26(b), the sensor remains highly responsive even as the respiratory cycles become rapid and the chest displacement amplitude decreases.

The spectral analysis shown in Fig. 26(d) reveals the presence of two distinct frequency peaks. The first peak appears at approximately 0.35 Hz, corresponding to normal respiratory activity. In contrast, a second dominant peak emerges at around 0.57 Hz, indicating a significant frequency shift associated with rapid breathing. This shift reflects an increased respiratory rate of approximately 34 BPM. In the context of epilepsy monitoring, this real-time frequency shift may serve as a potential indicator for identifying preictal respiratory disturbance. The ability of the system to clearly distinguish between a 0.13 Hz (Slow) and a 0.57 Hz (Rapid) peak demonstrates its high sensitivity to respiratory variations that may be associated with periictal physiological changes. Because the phase of the S21 parameter is inherently sensitive to sub-millimeter changes in the distance between the two chest-mounted sensors, the system can reliably capture the onset of respiratory distress even when movements are shallow or irregular.

By identifying these deviations relative to the subject’s baseline, the system can identify abnormal respiratory patterns that may be associated with seizure-related physiological changes, ongoing ictal activity, or postictal respiratory suppression. This high-sensitivity tracking confirms that the proposed skin-conformal UWB sensor is a suitable, low-cost tool for continuous wearable monitoring and integration into future clinical seizure detection and SUDEP risk assessment frameworks.

### Respiratory characteristics associated with seizure activity

The preceding section highlighted the dynamic variations in respiratory patterns that can occur under different physiological conditions. These variations provide a context for understanding how breathing is affected during epileptic events. Respiratory disturbances are among the most prominent physiological alterations associated with seizures, often manifesting as changes in rhythm, amplitude, or regularity before, during, or after the seizure episode^38–40^. To comprehensively describe these changes, respiratory activity surrounding a seizure is commonly classified into three stages: preictal, ictal, and postictal. Each stage exhibits characteristic alterations in breathing patterns that reflect the underlying autonomic and cortical disruptions. The following subsections provide a detailed discussion of respiratory dynamics during each stage, outlining typical deviations observed in clinical studies and their physiological basis.

#### Preictal respiratory disturbances

The preictal phase is the interval leading up to seizure onset, during which measurable physiological deviations may develop before any observable clinical or electrographic activity^38^. Respiratory abnormalities such as increased respiratory rate (tachypnea), decreased rate (bradypnea), or instability in rhythm can begin seconds to minutes before seizures^41^. These deviations reflect early autonomic dysregulation originating from abnormal cortical or subcortical activity^39^. Preictally, the respiratory rate may exceed the upper limit of normal resting values or decrease significantly below baseline^42^. Instability in amplitude, reduced respiratory sinus arrhythmia, and disrupted cardiorespiratory coupling have also been documented as early signatures^38^. Detecting these preictal abnormalities enables prediction of seizure onset and supports early warning systems designed to alert clinicians or caregivers before clinical symptoms appear^7^.

#### Ictal respiratory disturbances

During the ictal phase, when the seizure is actively occurring, respiratory abnormalities often intensify^40^. Many seizure types, particularly generalized tonic–clonic seizures, are associated with ictal apnea, shallow breathing, irregular respiratory cycles, or complete cessation of airflow^43^. This disturbance is driven by excessive neuronal discharge interfering with autonomic and brainstem respiratory pathways^39^. Ictal respiratory dysfunction may involve complete or partial apnea, significant reduction in tidal volume, marked irregularity in breathing pattern, and transient episodes of gasping or ataxic breathing^38^. These abnormalities can compromise oxygenation, increase the risk of hypoxia, and contribute to severe complications including SUDEP (Sudden Unexpected Death in Epilepsy)^44^. Continuous monitoring during the ictal phase provides crucial insight into seizure severity and autonomic involvement^6^.

#### Postictal respiratory disturbances

The postictal phase follows seizure termination and is frequently characterized by persistent respiratory instability^45^. Patients may experience prolonged apnea, shallow breathing, reduced respiratory drive, or difficulty restoring normal respiratory rhythm^43^. Postictal respiratory dysfunction is particularly significant due to its clinical association with oxygen desaturation, impaired arousal, and elevated SUDEP risk^44^. Common postictal features include slow or irregular breathing, prolonged periods of reduced airflow, delayed recovery of normal respiratory rhythm, and continued impairment in autonomic control^45^. Monitoring respiratory behavior in the postictal phase is essential for assessing recovery, preventing complications, and ensuring patient safety, especially when the patient is unconscious or unresponsive^4^.

#### Integration with the proposed UWB CPW sensor

In this work, the proposed UWB CPW-based sensing system continuously monitors phase variations in the transmission coefficient (S₂₁) to capture respiratory dynamics across all periictal stages^15^. Normal breathing produces stable, periodic phase fluctuations, while preictal, ictal, and postictal disturbances generate measurable deviations in rate, amplitude, and rhythm^16^. By identifying these deviations relative to the subject’s baseline, the system can be used to monitor abnormal respiratory patterns that may be associated with seizure activity, ongoing ictal activity, or postictal respiratory suppression^7^. Its skin-conformal design and high sensitivity make it suitable for wearable, continuous monitoring and for incorporation into future clinical seizure detection and SUDEP risk assessment frameworks^5^.

## Discussion and comparison

The main contribution of this work lies in the design and fabrication of a flexible ultra-wideband (UWB) CPW tattoo-like sensor using low-cost transparent PVC substrate and gold-leaf conductive material. The proposed structure achieves high flexibility, optical transparency, and ultra-low fabrication cost, making it suitable for wearable biomedical applications. In addition, the sensor is integrated into a chest-mounted TX–RX configuration for respiratory monitoring based on S₂₁ phase variations, enabling a non-contact and non-invasive sensing mechanism. Furthermore, a systematic experimental study was conducted to optimize sensor placement on the human chest for improved signal stability and sensing performance.

The proposed UWB CPW-based wearable sensor demonstrates stable electromagnetic behavior and reliable respiratory sensing performance. However, unlike conventional sensor systems designed for far-field radiation, the primary function of this structure is to ensure stable near-field coupling for body-centric sensing, where variations in the transmission coefficient (S₂₁) are used as the sensing mechanism. The parametric analysis of the ground-plane modifications ($\:{W}_{g1}$, $\:{L}_{g1}$, and $\:{L}_{g2}$) shows that impedance tuning is not only responsible for achieving wideband matching, but also plays a key role in controlling the stability of the near-field coupling environment. This directly influences the consistency of the S₂₁ phase response, which is the primary observable used for respiratory motion extraction. Mechanical robustness results indicate that bending and crumpling introduce only minor variations in the electromagnetic response, which confirms that the sensing mechanism is resilient to realistic deformation conditions expected in wearable operation. This behavior is consistent with previously reported flexible and conformal sensing platforms for on-body applications^22,36^.

Measured S₁₁ results show good agreement with simulations, with minor discrepancies at higher frequencies attributed to fabrication tolerances associated with gold-leaf conductive layers. Importantly, these variations do not significantly affect the stability of the S₂₁ phase used for respiration monitoring. SAR analysis confirms that all values remain well below IEEE safety limits at 20 dBm transmit power, ensuring safe operation for continuous wearable use in biomedical environments^46^.

Respiratory sensing experiments demonstrate that system performance is strongly dependent on the TX–RX spatial configuration. The 10 cm separation produces the most stable phase response due to an optimal balance between excessive near-field coupling (observed at 0 cm) and signal attenuation (observed at 20 cm). The front-to-back configuration introduces additional degradation due to propagation through heterogeneous tissue layers, resulting in reduced phase stability. These results highlight that sensing accuracy is governed by electromagnetic coupling conditions rather than conventional sensor radiation parameters^13^.

The proposed system offers several advantages, including ultra-wide bandwidth, mechanical flexibility, low-cost fabrication, optimized sensor placement, and low SAR compliance, making it suitable for wearable biomedical applications. However, certain limitations should be noted. The manual fabrication process using gold-leaf may introduce slight geometric inconsistencies affecting high-frequency impedance behavior. In addition, extreme bending or deformation can cause minor frequency shifts, although the sensing mechanism based on S₂₁ phase variations remains stable. Moreover, the current setup relies on a vector network analyzer (VNA), which limits portability and real-time deployment, motivating future integration with compact UWB transceiver systems.

When compared with recently reported wearable UWB CPW-based sensors (Table 3), the proposed system demonstrates a significantly wider operational bandwidth (2.4–17 GHz) while maintaining compact size and full flexibility. Unlike conventional designs that focus mainly on communication or radiation performance, the proposed approach is specifically optimized for near-field body-coupled sensing, where respiratory motion is directly translated into S₂₁ phase variations. This provides higher sensitivity to small chest displacements compared to amplitude-based sensing approaches used in conventional systems. Furthermore, recent high-quality studies were incorporated into Table 3 to provide a more comprehensive and updated comparison with state-of-the-art wearable sensing systems. Additionally, the use of transparent and low-cost materials distinguishes this work in terms of fabrication accessibility and scalability. Tables 3 and 4 presents a comparison between the proposed sensor and recently reported wearable UWB CPW-based designs in terms of key performance metrics, including physical size, sensor geometry, flexibility level, operating bandwidth, SAR compliance, and target biomedical application. This comparison highlights the main design and performance parameters used to evaluate and differentiate wearable sensing sensors in similar biomedical monitoring systems.

**Table 4:** Comparison of the proposed sensor with previously published work.

**Ref**	**Size (mm²)**	**Geometry/CPW**	**Flexibility**	**Bandwidth (GHz)**	**SAR (W/kg)**	**Application**
^7^	60 × 60	CPW monopole	Moderate	1.5–10	1.191(1 g), 1.39(10 g)	Vital signs &lung water
^19^	30 × 20	monopole	High	2.85–9.81	Not specified	WBAN, wearable monitoring
^47^	65 × 65	CPW feed	High	2.7–10.5	Not specified	Wearable biomedical
^48^	48 × 36	circular-shaped two-slot	High	3.1–10.7	Not specified	real-time monitoring of patient health
^49^	40 × 40	CPW feed	High	2.5–12	0.662 (1 g), 0.210(10 g)	Real-time breath monitoring
^50^	ultra-miniaturized	UWB patch	High	Wideband (UWB)	Not specified	e-health/vital sign monitoring
^51^	40 × 41	dual-band patch	High	Multi-band (2.4/5.8 GHz)	SAR evaluated, within limits	biomedical IoT monitoring
^52^	Compact	UWB antenna with integrated band-notch	Moderate	UWB	Not specified	WBAN applications
This work	40 × 50	CPW slot (tattoo-like transparent structure)	High, transparent, conformal	2.4–17	0.946(1 g), 0.258(10 g)	Respiratory + near-field S₂₁ phase-based sensing for potential pre-seizure monitoring

Future work will focus on extending the proposed system toward practical real-world healthcare applications. In this study, experimental validation has already been performed on two healthy subjects, demonstrating the feasibility of the proposed approach for respiratory monitoring. However, further evaluation on a larger population with different physiological and pathological conditions is required, particularly for epilepsy-related respiratory monitoring. In addition, future developments may integrate artificial intelligence and machine learning algorithms for automatic classification of abnormal breathing patterns and early seizure prediction. The proposed system can also be combined with Internet of Things (IoT) platforms or mobile healthcare applications for real-time remote monitoring, alert generation, and cloud-based data analysis. Furthermore, the integration of compact wireless modules and low-power electronics will enable the development of a fully standalone wearable biomedical monitoring device suitable for modern healthcare and industrial applications.

## Conclusion

A flexible, transparent tattoo-based CPW slot sensor has been presented for wearable biomedical monitoring, with a particular focus on respiratory-phase detection. The proposed design achieves a very wide operating bandwidth of 2.4–17 GHz and maintains stable performance under bending and crumpling, confirming its suitability for skin-mounted applications. Respiratory monitoring experiments demonstrated a clear and consistent breathing pattern, validating the sensor’s ability to capture real-time physiological variations at normal human breathing rates. Safety analysis further confirmed low SAR values, ensuring safe long-term operation on the body. Overall, the combination of ultra-thin transparent materials, strong mechanical durability, wide bandwidth, and reliable sensing performance highlights the potential of this sensor for continuous health monitoring and future integration with compact UWB transceiver systems. The proposed system was evaluated on volunteers to assess its repeatability and robustness under practical conditions. The recorded S21 phase variations consistently exhibited clear periodic breathing patterns across both participants, confirming stable respiratory detection performance. Although minor variations in amplitude and breathing rate were observed due to natural physiological differences and slight variations in sensor placement, the overall respiratory signatures remained clearly distinguishable, demonstrating reliable system behavior across different users. Furthermore, the dual-sensor configuration based on S21 phase tracking provides a robust mechanism for capturing subtle chest wall motion. The presence of multiple resonant modes within the UWB band enhances sensitivity to small physiological movements while maintaining stable performance under realistic on-body conditions. This multi-resonant and wideband response improves the reliability and consistency of respiratory monitoring in practical wearable scenarios. While the proposed system demonstrates reliable respiratory monitoring, further investigation involving clinical datasets and epileptic subjects is required to validate its applicability for seizure detection.

## Supplementary Information

Below is the link to the electronic supplementary material.

Supplementary Material 1

## Data Availability

All data generated or analyzed during this study are included in the published article. Dr Anwer is the corresponding author who can be contacted in order to access these data.
