# Supplementary figures and images for: Low-cost UWB CPW microwave tattoo sensor for respiratory monitoring using near-field phase variation
Source: Sci Rep. 2026 Jun 30;16:19942. doi: 10.1038/s41598-026-58536-5 (PMC13319781; doi:10.1038/s41598-026-58536-5)

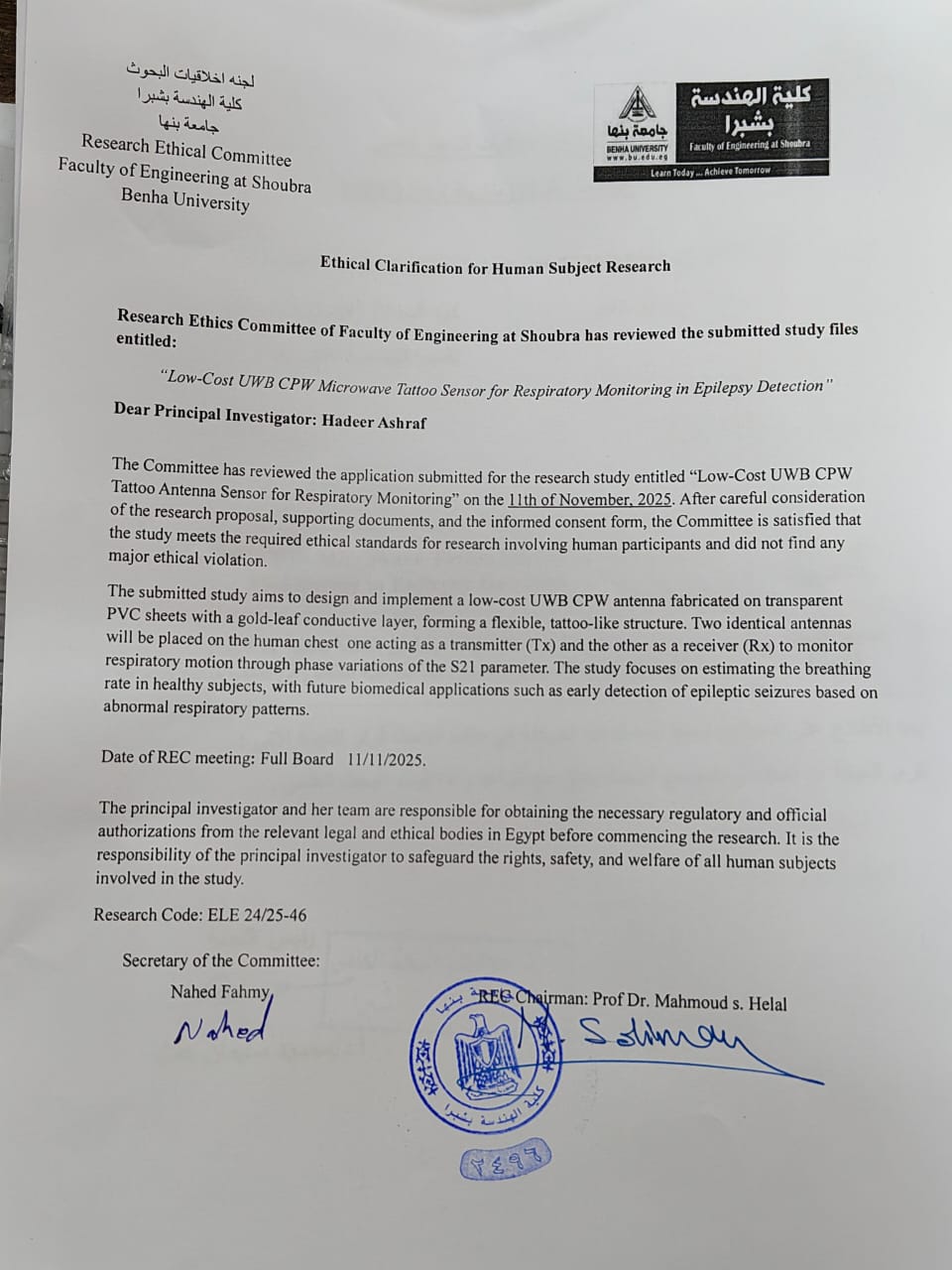

Supplement: Supplementary file 1 — Supplementary Material 1 [file 41598_2026_58536_MOESM1_ESM.jpeg]
